# Interhemispheric co-alteration of brain homotopic regions

**DOI:** 10.1007/s00429-021-02318-4

**Published:** 2021-06-25

**Authors:** Franco Cauda, Andrea Nani, Donato Liloia, Gabriele Gelmini, Lorenzo Mancuso, Jordi Manuello, Melissa Panero, Sergio Duca, Yu-Feng Zang, Tommaso Costa

**Affiliations:** 1 Department of Psychology, GCS-fMRI, Koelliker Hospital, University of Turin, Turin, Italy; 2 Department of Psychology, FOCUS Lab, University of Turin, Turin, Italy; 3 Center for Cognition and Brain Disorders, Institutes of Psychological Sciences, Hangzhou Normal University, Hangzhou, 311121 China; 4 Zhejiang Key Laboratory for Research in Assessment of Cognitive Impairments, Hangzhou, 311121 China

**Keywords:** Brain alterations, Voxel-based morphometry, Pathological co-alteration, Alzheimer’s disease, Schizophrenia, Depressive disorder

## Abstract

Asymmetries in gray matter alterations raise important issues regarding the pathological co-alteration between hemispheres. Since homotopic areas are the most functionally connected sites between hemispheres and gray matter co-alterations depend on connectivity patterns, it is likely that this relationship might be mirrored in homologous interhemispheric co-altered areas. To explore this issue, we analyzed data of patients with Alzheimer’s disease, schizophrenia, bipolar disorder and depressive disorder from the BrainMap voxel-based morphometry database. We calculated a map showing the pathological homotopic anatomical co-alteration between homologous brain areas. This map was compared with the meta-analytic homotopic connectivity map obtained from the BrainMap functional database, so as to have a meta-analytic connectivity modeling map between homologous areas. We applied an empirical Bayesian technique so as to determine a directional pathological co-alteration on the basis of the possible tendencies in the conditional probability of being co-altered of homologous brain areas. Our analysis provides evidence that: the hemispheric homologous areas appear to be anatomically co-altered; this pathological co-alteration is similar to the pattern of connectivity exhibited by the couples of homologues; the probability to find alterations in the areas of the left hemisphere seems to be greater when their right homologues are also altered than vice versa, an intriguing asymmetry that deserves to be further investigated and explained.

**Supplementary Information:**

The online version contains supplementary material available at 10.1007/s00429-021-02318-4.

## Introduction

Brain asymmetries (both structural and functional) are frequently found in humans and animals and result in typical hemispheric specializations. These asymmetries are supposed to be caused by a panoply of factors, such as hereditary, developmental, evolutionary, experiential and pathological ones. In particular, evolutionary processes might have shaped the brain so as to favor specialization over the mere duplication of structures. In addition, hemispheric differentiation might be induced by asymmetrical ways of acting as well as by mechanisms of brain plasticity triggered by experience (Toga and Thompson 2003). This is likely to be related to the Hebbian mechanism (Hebb 1949), and, more generally, to the processes that subserve the phenomenon of structural covariance, which are thought to include genetic influences, normal development and aging, as well as pathological effects (Evans 2013).

The development of pathological processes might exacerbate existing brain asymmetries, due to the asymmetrical progression pattern of some diseases. Indeed, evidence shows that a number of pathological conditions mainly affect the left hemisphere (Minkova et al. 2017). In particular, longitudinal studies show a more rapid left hemisphere cortical deterioration in patients with Alzheimer’s disease (AD), which is typically characterized by a progressive gray matter (GM) loss that originates in temporo-parietal and entorhinal cortices, subsequently spreading toward the frontal lobe and eventually to the sensorimotor sites (Thompson et al. 2001, 2003). After approximately 2 years, the right hemisphere appears to deteriorate with a similar pattern.

Mild cognitive impairment follows a similar, albeit less severe, course. In both AD and mild cognitive impairment, the decline of the left hemisphere is associated with lower scores in language-based neuropsychological tests (Derflinger et al. 2011). Furthermore, patients with Huntington’s disease show asymmetrical GM alterations, frequently characterized by a more significant atrophy of the left striatum during the pre-symptomatic stage of the disease (Lambrecq et al. 2013).

With regard to AD neurodegeneration, asymmetries suggest either that the right hemisphere is less susceptible than the left to this disorder, or that the pathological process causes in the left hemisphere more severe metabolic deficits as well as structural alterations (Loewenstein et al. 1989). On the other hand, with regard to the healthy aging condition, it has been consistently observed a tendency for a faster GM reduction in the left prefrontal cortex compared to the right one (Thompson et al. 2003).

The apparent greater susceptibility of the left hemisphere to several brain disorders is as yet unexplained. A possible cause could be the dominance of that hemisphere for important cognitive functions, such as language (Frost et al. 1999; Josse and Tzourio-Mazoyer 2004; Springer et al. 1999; Vigneau et al. 2006) and motor control (Janssen et al. 2011; Serrien and Sovijarvi-Spape 2015; Taylor and Heilman 1980), which might induce excitotoxicity due to the more intense neuronal activity (Jagust 2009). On the other hand, visuospatial functions have been predominantly associated with right hemisphere processes (Duecker et al. 2013; Gotts et al. 2013; Nielsen et al. 2013; Sturm and Willmes 2001). However, few investigations have focused on interhemispheric atrophic differences. Furthermore, the reduction of GM volume does not only entail specific lateralized systems, but frequently outspreads across cortical and subcortical areas. A recent meta-analysis by Minkova et al. (2017) provides further evidence against the greater susceptibility of the left hemisphere to neuropathology. Although GM reductions tend progressively to be asymmetric, the study found no evidence for an increased vulnerability of the left hemisphere.

It has recently been found that GM alterations caused by different brain diseases do not spread randomly, but are distributed according to specific co-alteration patterns which are often characterized by a network-like architecture, depending on both structural and functional connectivity (Cauda et al. 2012, 2017, 2018a, b; Crossley et al. 2014, 2015, 2016; Fornito et al. 2015; Manuello et al. 2018; McTeague et al. 2016; Menon 2013; Raj et al. 2012; Saxena and Caroni 2011; Seeley et al. 2009; Yates 2012; Zhou et al. 2012). Apart from pathology-specific patterns, converging evidence suggests that an important group of co-altered areas is often affected by many brain disorders (Baker et al. 2014; Cauda et al. 2017, 2018a; Douaud et al. 2014; Ellison-Wright and Bullmore 2010; Etkin and Wager 2007; Goodkind et al. 2015; Hamilton et al. 2012; Jagust 2013; Menon 2013; Saxena and Caroni 2011). In other words, the variety of structural alterations produces typical patterns in which some brain areas appear to be not only more altered but also more specifically affected than others (Cauda et al. 2019; Liloia et al. 2018).

The study of cerebral asymmetries, specifically the ones related to the entity and progression of brain diseases, can shed new light on how GM co-alterations are distributed between the hemispheres (i.e., interhemispheric spread). We can argue that (i) since the homotopic areas are usually the most functionally connected sites between hemispheres (Biswal et al. 1995; Cauda et al. 2011b; Lowe et al. 1998; Medvedev 2014; Raemaekers et al. 2018; Salvador et al. 2005a, b; Stark et al. 2008; Toro et al. 2008), and (ii) since the GM co-alterations—based on statistical associations between alterations across several brain areas—partly depend on standard connectivity patterns (Cauda et al. 2018a, b; Manuello et al. 2018; Tatu et al. 2018), it is likely that this statistical relationship might be mirrored in interhemispheric co-altered areas that are anatomically homologue. Probably, alterations in homologous areas entail statistical interdependence, which can be interpreted as a tendency to co-alter. It is also probable that not only these co-altered areas express statistical interdependence but that, similarly to the anatomical asymmetries occurring in the interhemispheric alterations, asymmetries between co-altered areas may occur in their statistical relationship (Patel et al. 2006). In other words, for certain couples of homologous brain areas, say A in the right hemisphere and B in the left hemisphere, the probability of being altered of A given the alteration of B [P(A|B)] may be higher than the probability of being altered of B given the alteration of A [P(A|B)], or vice versa. A result in favor of one alternative would imply a tendency in the conditional probability, and this could be seen in terms of a higher probability to find an alteration in a certain area, given the alteration of its contralateral homologue (Patel et al. 2006). We could infer, therefore, the preferential directionality of the alteration spread (Patel et al. 2006).

In light of these premises, our study aims at answering the following important issues. (1) Does a statistical relationship occur between the anatomical alterations of homologous areas caused by brain diseases and, if so, how strong is this relationship? (2) Similarly to what happens in the pattern of GM alterations that seems to be influenced by brain connectivity, can the pathological co-alteration of homologous areas be influenced by brain connectivity patterns? (3) Finally, in case of a significant co-alteration between homologous areas, can the directionality of their conditional probability of being co-altered be obtained?

To answer question (1) we used GM alteration data from the BrainMap database (Fox et al. 2005; Fox and Lancaster 2002; Laird et al. 2005); these data were analyzed with an innovative technique that allows to calculate a map showing the pathological homotopic anatomical co-alteration (PHAC) between homologous brain areas. PHAC map was then statistically compared with the meta-analytic homotopic connectivity (MHC, Mancuso et al. 2019) map obtained from the functional database of BrainMap and calculated using the same algorithms applied for creating the PHAC map. This method allowed us to address question (2), because it offers a meta-analytic connectivity modeling (MACM) map (Cauda et al. 2011a; Robinson et al. 2010) between homologous areas that is meta- analytically tantamount to the voxel-mirrored homotopic connectivity (VMHC) (Guo et al. 2013; Li et al. 2015; Wang et al. 2015a, b; Zuo et al. 2010). Finally, to answer question (3) we applied an empirical Bayesian technique, the Patel’s *τ* (Patel et al. 2006), so as to determine a preferential directional PHAC (dPHAC) on the basis of the possible tendencies (i.e., directionalities) in the conditional probability of being co-altered of homologous brain areas.

These analyses have been carried out both on the VBM data set disease-related of BrainMap and on the four of the most represented brain disorders of this data set [schizophrenia (SCZ), Alzheimer’s disease (AD), bipolar disorder (BD) and depressive disorder (DD)]. The rationale for our approach was based on the opportunity to take advantage of the possible greatest amount of data as well as on recent theoretical views that demand a neurobiological understanding to better assess how the brain reacts to neurological and psychiatric conditions (Buckholtz and Meyer-Lindenberg 2012; Fornito et al. 2015; Gandal 2018; Goodkind et al. 2015; Iturria-Medina and Evans 2015; McTeague et al. 2016; Raj et al. 2012; Sprooten et al. 2017; Zhou et al. 2012).

## Materials and methods

### Selection of studies

The pool of all eligible neuroimaging experiments was retrieved from the BrainMap database (http://brainmap.org/) (Fox et al. 2005; Fox and Lancaster 2002; Laird et al. 2009, 2005; Vanasse et al. 2018) using a Sleuth query. BrainMap is an online open access database that uses a systematic coding scheme which contains over 15,000 published human neuroimaging experimental results and reports over 120,000 brain locations in stereotactic space. The main division of this database is between voxel-based morphometry (VBM) and functional data. For our meta-analysis, both the VBM and functional data sets have been used. First, using the BrainMap software application ‘Sleuth 2.4’ we queried the VBM BrainMap database (January 2018) using the following search criteria:

1. decreases: (experiments context is disease) AND (experiment contrast is gray matter) AND (experiments observed changes is controls > patients);
2. increases: (experiments context is disease) AND (experiment contrast is gray matter) AND (experiments observed changes is patients > controls).

We retrieved 994 experiments (i.e., 994 sets of alteration stereotactic coordinates indicating the foci of significant case–control alterations). Then the retrieved data set was codified on the basis of the ICD-10 classification (World Health Organization 1992) by an expert researcher. In addition, all the eligible articles were analyzed by two expert researchers to ascertain that they satisfied the following inclusion criteria: (a) to be an original work published in a peer-reviewed English language journal; (b) to include a whole-brain VBM analysis; (c) to include a comparison between pathological sample and healthy control participants; (d) to report GM decrease/increase changes in pathological sample; (e) to adopt a specified VBM analysis; f) to report the locations of GM changes (specifically cartesian coordinates in a standardized 3D space) in a definite stereotactic space (i.e., Talairach/Tournoux or Montreal Neurological Institute). On the grounds of the aforementioned criteria, 793 articles were included (585 of GM decreases and 208 of GM increases), for a total of 1361 experiments (980 of GM decreases and 381 of GM increases) and 29,403 subjects. Descriptive information of interest was extracted from each full-text article. Since some of the foci coordinates were reported in MNI space while other in Talairach space, locations reported in MNI were converted into Talairach space using Lancaster’s icbm2tal transform, following the approach of Laird et al. (2010) and of Lancaster et al. (2007). The complete overview of the selection process is reported in Table 1. More detailed information about the description and distribution of the VBM data set disease-related included in our meta-analysis are viewable in the Supplementary Table S1. Table S2 shows the sample characteristics for the four most represented brain disorders in the BrainMap VBM database (i.e., SCZ, AD, BD and DD).

**Table 1:** Synopsis of the selection procedure with number of articles identified at each stage

BrainMap identification		Screening	Eligibility	BrainMap included	
Morphological (VBM) records	Functional records			Morphological (VBM) records	Functional records
Articles 994 ⇓ Additional records 0	Articles 2376 ⇓ Additional records 0	Abstract exclusions Eligibility for full-text lecture	Full-text exclusions Selected studies 793 VBM 2376 Functional	Selected studies 585 GM decrease 208 GM increase Sample (*N*) 29,403 ⇓ SCZ (114 studies) AD (55 studies) DD (54 studies) BD (46 studies) Others (524 studies)	Selected studies 2376 Selected experiments 13,148 Sample (*N*) 68,152
Phase 1 ⇓ data search	Phase 2 ⇓ data search	Phase 3	Phase 4	Phase 5 ⇓ data extraction	Phase 6 ⇓ data extraction

Finally, we did a systematic search on the functional data set of BrainMap using the following search criteria:

(1) (experiments context is normal mapping) AND (experiments activation is activations only) AND (subjects diagnosis is normals).

We retrieved 2376 articles, for a total of 13,148 experiments, 110 paradigm classes and 68,152 subjects. All the retrieved experiments were used for the subsequent MHC (Mancuso et al. 2019) analysis (see also Table S3 in Supplementary Material)**,** after the conversion of the coordinates in Talairach space.

Authors declare to have signed a written agreement with the BrainMap group and the University of Texas, San Antonio, USA, so as to have access to the BrainMap database.

We adopted the definition of meta-analysis accepted by the Cochrane Collaboration (Green et al. 2008) and performed the process of selecting eligible articles according to the ‘PRISMA Statement’ international guidelines (Liberati et al. 2009; Moher et al. 2009) [see Figure S1 (PRISMA flow chart) in the online Supplementary Material].

### Anatomical likelihood estimation and creation of the modeled activation map

We employed the anatomical likelihood estimation (ALE) (Eickhoff et al. 2009, 2012; Turkeltaub et al. 2012) so as to construct the maps to feed the PHAC and Patel’s algorithms. The ALE is a quantitative voxel-based meta-analysis that can provide information about the anatomical reliability of results. It compares the results with a sample of reference studies obtained from the existing literature. Every focus of each experiment is considered to be the central point of a three-dimensional Gaussian probability distribution:

The ALE is a quantitative voxel-based meta-analysis that can provide information about the anatomical reliability of results. It compares the results with a sample of reference studies obtained from the existing literature. Every focus of each experiment is considered to be the central point of a three-dimensional Gaussian probability distribution:

1 $$ p\left( d \right) = \frac{1}{{\sigma ^{3} \sqrt {\left( {2\pi } \right)^{3} } }}e^{{\frac{{ - d^{2} }}{{2\sigma ^{2} }}}} $$

in which *d* represents the Euclidean distance between the voxels and the focus taken into account, whereas σ represents the spatial uncertainty. The standard deviation is calculated through the full-width at half-maximum (FWHM) with the following formula:

2 $$ \sigma  = \frac{{FWHM}}{{\sqrt {8ln2 } }} $$

which results in different values of σ and thus in modeled activation or alteration (MA) maps with different size for each experiment according to their number of subjects.

The MA maps are derived from a Gaussian probabilistic cloud for each focus. If the focus is close to the brain median line, then the probabilistic cloud may extend for few millimeters in both the hemispheres, thus producing spurious co-alteration/coactivation results. To address this potential issue, we adjusted the offset values that were close to the median line. By taking into consideration the mean spatial uncertainty that is typical of these meta-analytic data (Eickhoff et al. 2009), we expected that on average the Gaussian cloud may extend around 12 mm, so we modeled a sphere having a mean radius of 12 mm and compensated for the probabilistic cloud extension an area of 12 mm both on the left and on the right of the median line; to do so, we applied a weight decreasing function with distance in millimeters between the median line $i$ and the voxel $j$ taken into account, proportional to $\frac{1}{{d_{{ij}} }}$, which attributes to the voxels major or minor activations according to their proximity to the median line.

### Maps of pathological homotopic anatomical co-alteration and the calculation of the conditional probability unbalance

To determine the PHAC maps we conceived a novel method allowing us to construct a map of the homotopic anatomical co-alterations using meta-analytic data. This method can identify if the anatomical alteration of a cerebral area statistically co-occurred with the alteration of its homologue in the contralateral hemisphere. With this analysis we can therefore construct a PHAC map, in which values are assigned proportionally to the statistical relationship between cerebral regions of one hemisphere and their contralateral homologues.

The brain has been symmetrically partitioned by means of an anatomical atlas obtained from the Talairach atlas extracted from the Talairach Daemon (Lancaster et al. 1997, 2000; http://talairach.org/). The atlas was co-registered to the same 2 mm resolution GingerALE standard of the MAs maps (http://brainmap.org/ale/colin_tlrc_2x2x2.nii.gz) using FLIRT from FSL (Smith et al. 2004; http://www.fmrib.ox.ac.uk/fsl/). To produce symmetric maps of homotopic co-alteration, the atlas was subsequently symmetrized by substituting the left hemisphere with a copy of the right one flipped along the midline. To construct the PHAC map, we created an alteration matrix with the couples of homologous areas as nodes. In a *N* × *M* matrix every row indicates an experiment, whereas every column indicates a node corresponding to an area of the brain; in our case, the numbers of experiments (functional and VBM data) × 1105 nodes constitute the matrix. For every experiment, a node was considered to be altered if the MA map (thresholded at *p* = 0.05) of the experiment reported 20% or more of the voxels of interest (VOIs) within the node. As showed in Mancuso et al. (2019), the arbitrary percentage of 20% of altered voxels, which is needed to consider a VOI as altered, does not bias the results and was showed to be a reasonable middle ground between 0%, which is obviously too liberal, and 40%, which can be argued to be too conservative.

From the *N* × *M* matrix we obtained the co-alteration strength between the homotopic nodes using the Patel’s κ index (Patel et al. 2006), thus generating the probability distribution of joint alteration occurrences for every couple of nodes. Specifically, given two nodes (*a* and *b*), it is possible to calculate the probability of all the possible conditions: (i) *a* and *b* are both altered; (ii) neither *a* nor *b* is altered; (iii) *a* is altered but not *b*; (iv) *b* is altered but not *a* (Table 2). Frequencies of these cases throughout the experiments result in the following four states of probabilities:

$$ \theta _{1}  = P\left( {a = 1,b = 1} \right) $$

$$ \theta _{2}  = P\left( {a = 0,b = 1} \right) $$

$$ \theta _{3}  = P\left( {a = 1,b = 0} \right) $$

$$ \theta _{4}  = P\left( {a = 0,b = 0} \right) $$

**Table 2:**
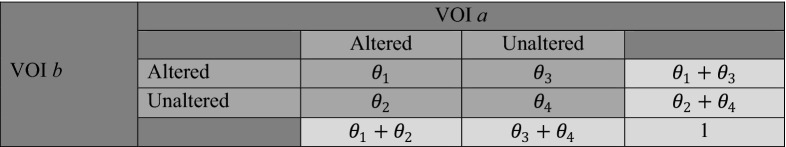
Marginal probabilities between altered and unaltered volumes of interest (VOIs)

These states of probabilities represent the conjoint state frequencies of a couple of nodes (*a* and *b*) in their four possible combinations. The following table illustrates the marginal probabilities:

Considering these four probabilities, we can apply the two indices κ and *τ* of Patel et al. (2006) for determining connectivity and directionality, respectively. These two indices have been shown to be effective with simulated data by Smith et al. (2011). However, with regard to the Patel’s *τ*, Wang et al. (2017) have criticized its usefulness. It should be observed that the criticism by Wang et al. focuses on issues (i.e., deconvolution of the hemodynamic response and temporal resolution) that are associated with the application of empirical Bayesian techniques to fMRI data; however, this is not the case of the present study, which takes into account morphometric data derived from the scientific literature.

The Patel’s κ is capable of measuring the probability that two nodes (*a* and *b*) are co-altered with respect to the probability that *a* and *b* are independently altered. Patel’s κ is defined as follows:

3 $$ \kappa  = \left( {\vartheta _{1}  - E} \right)/\left[ {D\left( {\max \left( {\vartheta _{1} } \right) - E} \right) + \left( {1 - D} \right)\left( {E - \min \left( {\vartheta _{1} } \right)} \right)} \right] $$

where

$$ E = \left( {\vartheta _{1}  + \vartheta _{2} } \right)\left( {\vartheta _{1}  + \vartheta _{3} } \right) $$

$$ \max \left( {\vartheta _{1} } \right) = \min \left( {\vartheta _{1}  + \vartheta _{2} ,\vartheta _{1}  + \vartheta _{3} } \right) $$

$$ \min \left( {\vartheta _{1} } \right) = \max \left( {0,2\vartheta _{1}  + \vartheta _{2}  + \vartheta _{3}  - 1} \right) $$

$$ D = \left\{ {\begin{array}{*{20}c}    {\frac{{\theta _{1}  - E}}{{2\left( {\max \left( {\theta _{1} } \right) - E} \right)}} + 0.5,\quad\,{\text{if}}\,\theta _{1}  \ge E}  \\    {0.5 - \frac{{\theta _{1}  - E}}{{2\left( {E - \min \left( {\theta _{1} } \right)} \right)}},\quad{\text{otherwise}}}  \\   \end{array} } \right. $$

In the fraction, the numerator is the difference between the probability that *a* and *b* are altered together and the expected probability that *a* and *b* are independently altered, whereas the denominator is a weighted normalizing constant. ${\text{Min}}\left( {\vartheta _{1} } \right)$ stands for the maximum value of conjoint probability $P\left( {a,b} \right)$, given $P\left( a \right)$ and $P\left( b \right)$, while ${\text{max}}\left( {\vartheta _{1} } \right)$ stands for the minimum value of $P\left( {a,b} \right)$, given $P\left( a \right)$ and $P\left( b \right)$. Patel’s κ values range from –1 and 1. A value of |κ| that is close to 1 indicates high connectivity. Patel’s κ statistical significance is evaluated by simulating with a Monte Carlo algorithm, a multinomial, generative model of data, which can consider the alterations of all the nodes. The Monte Carlo method obtains an estimate of posterior probability using the multinomial model as likelihood:

$$ p\left( {z|\theta } \right)\mathop \prod \limits_{{i = 1}}^{4} \theta _{i}^{{z_{i} }} $$

where $z_{i}$ are the sum of the respective $\theta _{i}$ of all experiments, that is, the number of times the given couple of nodes is co-altered, and a Dirichlet prior:

$$ p\left( {\theta |\alpha } \right) \propto \mathop \prod \limits_{{i = 1}}^{4} \theta _{{i_{i} }}^{{\alpha _{{j - 1}} }} $$

with $\theta _{i}  \ge 0$ and $\sum\nolimits_{{i = 1}}^{4} {\theta _{i} }  = 1.$ Then, the posterior distribution *p*(ϑ|z) is a Dirichlet with parameter $\gamma _{i}  = \alpha _{i}  + z_{i}  - 1$ with $i = 1 \ldots 4.$

The Monte Carlo samples from the posterior Dirichlet distribution 5000 random values and calculate the proportion of the samples in which κ > *e*, where *e* is the threshold of significance, set to 0.01. If this proportion is superior to 0.95 (*p* = 0.05), the edge is considered to be significant. This calculation has been run independently for each data set. To validate the Patel’s κ beyond any reasonable doubt, in the supplementary material is present a simulation of an (extremely unlikely) case that could produce false positives, showing that our methodology holds true even in worst case scenarios. Once the Patel’s κ of a couple of areas was calculated, such value was assigned to all the voxels of those two areas to obtain a PHAC map.

The *τ*(*a*, *b*) index, in turn, is capable of measuring how the alteration of node *a* influences the alteration of node *b*. The *τ* is calculated only on those edges that reached the statistical significance during the κ calculations. Thus, if two nodes *a* and *b* are significantly co-altered, the Patel’s *τ* indicates the directionality of the edge between them. Patel’s *τ* values range from –1 to 1. Positive values denote the influence of *a* over *b*, whereas negative values denote the influence of b over a. The *τ* index is defined as:

4 $$ \tau \left\{ {\begin{array}{*{20}c}    {1 - \frac{{\left( {\vartheta _{1}  + \vartheta _{3} } \right)}}{{\left( {\vartheta _{1}  + \vartheta _{2} } \right)}},\quad\,{\text{if}}\,\vartheta _{2}  \ge \vartheta _{3} }  \\    {\frac{{\left( {\vartheta _{1}  + \vartheta _{2} } \right)}}{{\left( {\vartheta _{1}  + \vartheta _{3} } \right)}} - 1,\quad{\text{otherwise}}}  \\   \end{array} } \right. $$

This index allows to obtain a value of directionality between two nodes and is thresholded using the threshold obtained before calculating the κ metrics. In other words, if we look at Table 2, the numerator and the denominator of *τ* are the marginal probability of the altered condition of the node *a* independently from the condition of the node *b*, and the marginal probability of the altered condition of the node *b* independently of the condition of the node *a*. This ratio of marginal probabilities gives a measure of alterations of two nodes and allows to estimate the directionality of alterations’ distribution, on the basis of the hypothesis is that if node *a* is the origin of a pathological spread toward node *b*, then node *a* is more likely to be found altered in many groups, both in co-alteration with node *b* (presumably in the groups of patients with a more advanced pathological development), and on its own. In contrast, node *b* may not be frequently altered in patients with an early pathological development and, when altered, it may almost always occur in co-alteration with node *a*. The Patel’s *τ* was used on the VBM and functional databases, to calculate two directed PHAC (dPHAC) maps, one for the decreases and one for the increases.

As explained before, we calculate the co-occurrence of alterations in every experiment, each at a time. If there are many foci distributed across different papers, for example because more studies are related to a specific pathology, this may improve the sensitivity of our method for this pathology and not produce false positives, as the permutation for the threshold of this part would consider the amount of data. If, on the contrary, the number of foci were greater on one side in the same paper, this would not bias the result, as the contingency table would have always the same value: 1. Let us make an example by considering two nodes (A and B), and two experiments, one of which has few foci in A and many foci in B, but both nodes have a significant (albeit different) ALE value. The contingency table would have 1 because both nodes are altered, though with different intensity. Even in the case of an experiment reporting a balanced number of foci and significant ALE values of A and B, the contingency table would have again 1. In other words, the calculus of the joint probabilities does not consider the intensity of the ALE values but is based only on the fact that an area results or not in being altered. It worth noting that such consideration applies when the number of foci is uneven between two homotopic regions, but not when both nodes have very few foci. In this case, the statistical power of our technique will drop, as noted in the paragraph “Unbalances in the directionality of the conditional probability among pathological homologous areas” of the discussion section.

Finally, the reliability towards subsampling of the PHAC and dPHAC maps was tested through a bootstrap procedure with 5000 iterations (see the Supplementary Materials for a detailed explanation and Figure S4).

### Calculation of the meta-analytic voxel-mirrored homotopic connectivity and AAL PHAC maps

For the meta-analytic calculus of the MHC map, we worked on the BrainMap functional database by applying to the data the same methodology used to construct the PHAC map (i.e., Patel’s κ index between brain homologous areas). Finally, we correlated the MHC map with the PHAC map. Similarly, we calculated the Patel’s *τ* to obtain a directed MHC (dMHC) map. In addition, the PHAC and MHC maps were also calculated using the AAL atlas (Tzourio-Mazoyer et al. 2002) to further confirm our results using a different parcellation.

### Large-scale network-based decompositions

Biswal et al. (2010) parcellated the brain surface using a large cohort of 1414 volunteers, who underwent a resting-state fMRI scan. The study found that in the brain at rest 20 large-scale networks can be identified; these networks are also identifiable when the brain is engaged in a task (Laird et al. 2011; Smith et al. 2009). On the basis of Biswal’s parcellation, we determined the mean ALE values of the GM voxels included in each of these 20 networks.

### Bias estimation

To ascertain whether or not the data may have a publication bias due to studies with more liberal thresholds, we used the jackknife technique (Tukey 1958), which is capable of determining a quadratic error parameter about the validity of each experiment in our database. To do so, we calculated the ALE with all the experiments $\left( {S_{0} } \right)$, then we recalculated the ALE by removing one experiment at a time $\left( {S_{k} } \right)$, with $k = 1,2,..,n$, where *n* is the total number of experiments. From this series of ALE maps, we calculated with regard to all the voxel *i* the sum of the quadratic difference between the total ALE and those obtained with the jackknife, as follows:

$$ E_{k}  = \mathop \sum \limits_{i} \left( {S_{{ik}}  - S_{{i0}} } \right)^{2} $$

This function of the quadratic error is minimal when $S_{k}$ tends to $S_{0}$.

## Results

These analyses have been carried out on the whole disease-related downloaded VBM data set (altogether) and on the four of the most represented brain disorders of this data set (SCZ, AD, BD, and DD).

### Pathological homotopic anatomical co-alteration

**Fig. 1:**
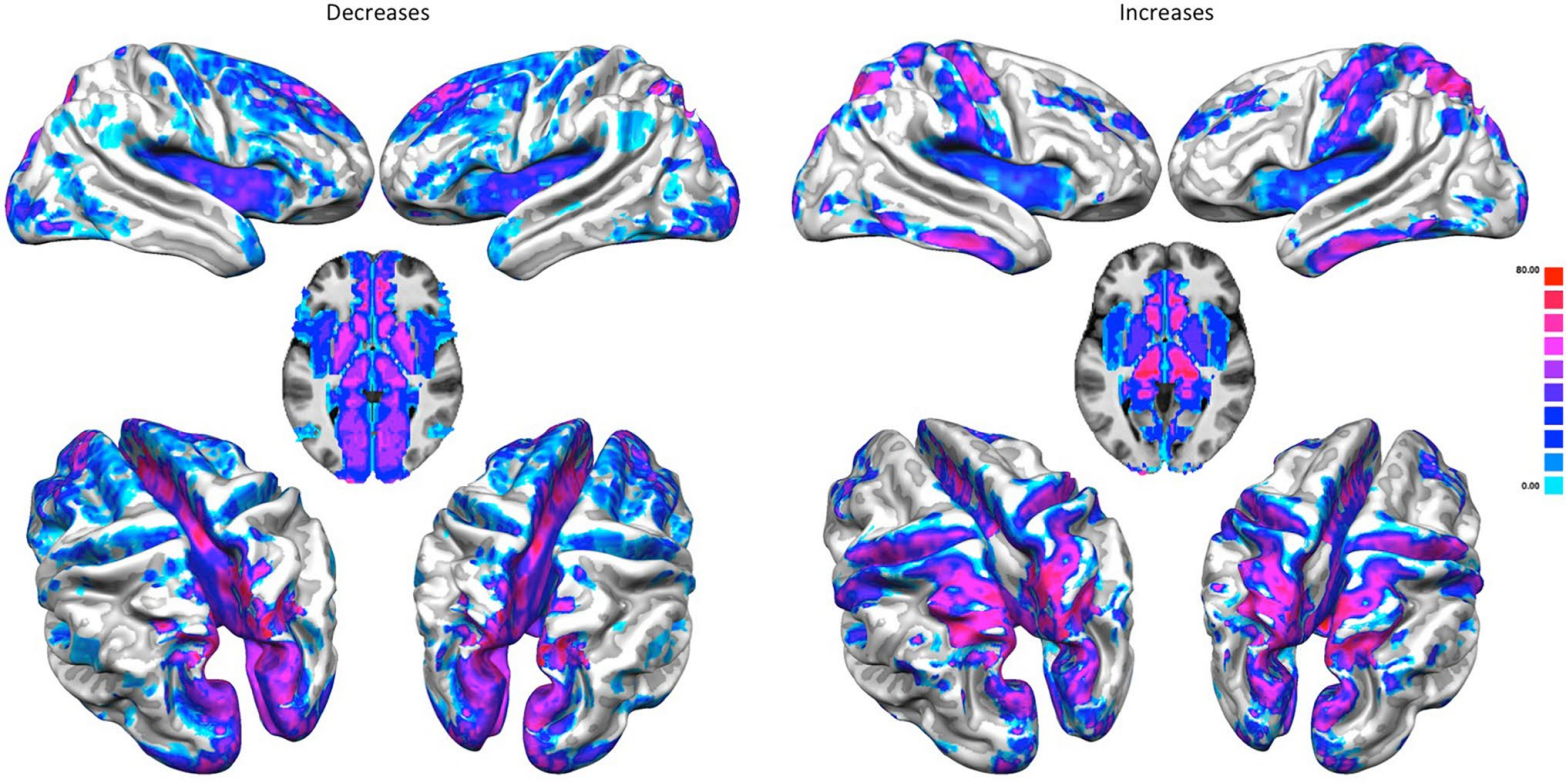
The pathological homotopic anatomical co-alteration (PHAC). The left panel shows the decrease-related PHAC, while the right panel shows the increase-related PHAC. Colors from blue to red indicate higher PHAC values. The κ values were multiplied by 100

The increase PHAC is characterized by co-alterations distributed in the upper and middle frontal gyrus, somatosensory, somatomotor, insular, posterior parietal, inferior temporal, cuneal, middle and anterior cingulate, thalamic, caudate and putaminal brain areas (see the right panel of Fig. 1). Although the decrease PHAC shows commonalities with the increase PHAC (encompassing insular, cuneal, cingulate, somatomotor, thalamic and striatal areas), it appears to be much more distributed and stronger in midline, thalamic, striatal and prefrontal brain areas (see the left panel of Fig. 1).

**Fig. 2:**
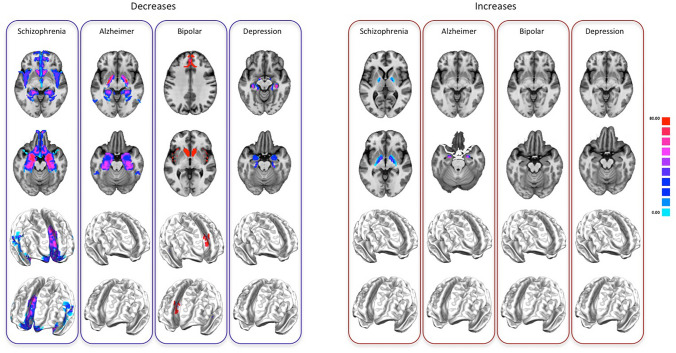
The pathological homotopic anatomical co-alteration (PHAC) of the four most represented brain diseases in BrainMap [Alzheimer’s disease (AD), schizophrenia (SCZ), bipolar disorder (BD), depressive disorder (DD)]. The left panel shows the decrease-related PHAC, while the right panel shows the increase-related PHAC. Colors from blue to red indicate higher PHAC values. The κ values were multiplied by 100

The analysis of the PHAC associated with the four of the most represented diseases in BrainMap (SCZ, AD, BD, DD) reveals that SCZ is characterized by a decrease PHAC encompassing insular, middle and anterior cingulate, middle prefrontal, superior temporal, postcentral, hippocampal, parahippocampal, orbitofrontal, caudate and amygdalar areas. On the other hand, the increase PHAC of SCZ principally involves the globus pallidus. AD is characterized by a decrease PHAC encompassing mainly posterior parietal areas, as well as the globus pallidus and hippocampus/parahippocampus. The increase PHAC of AD appears to involve exclusively the amygdala. BD and DD show only decrease PHACs. The PHAC of BD encompasses essentially anterior cingulate, insular cortices and caudate areas; while the PHAC of DD include amygdalar and hippocampal/parahippocampal areas (see Fig. 2, right panels for increase-related data and left panels for decrease-related data).

**Fig. 3:**
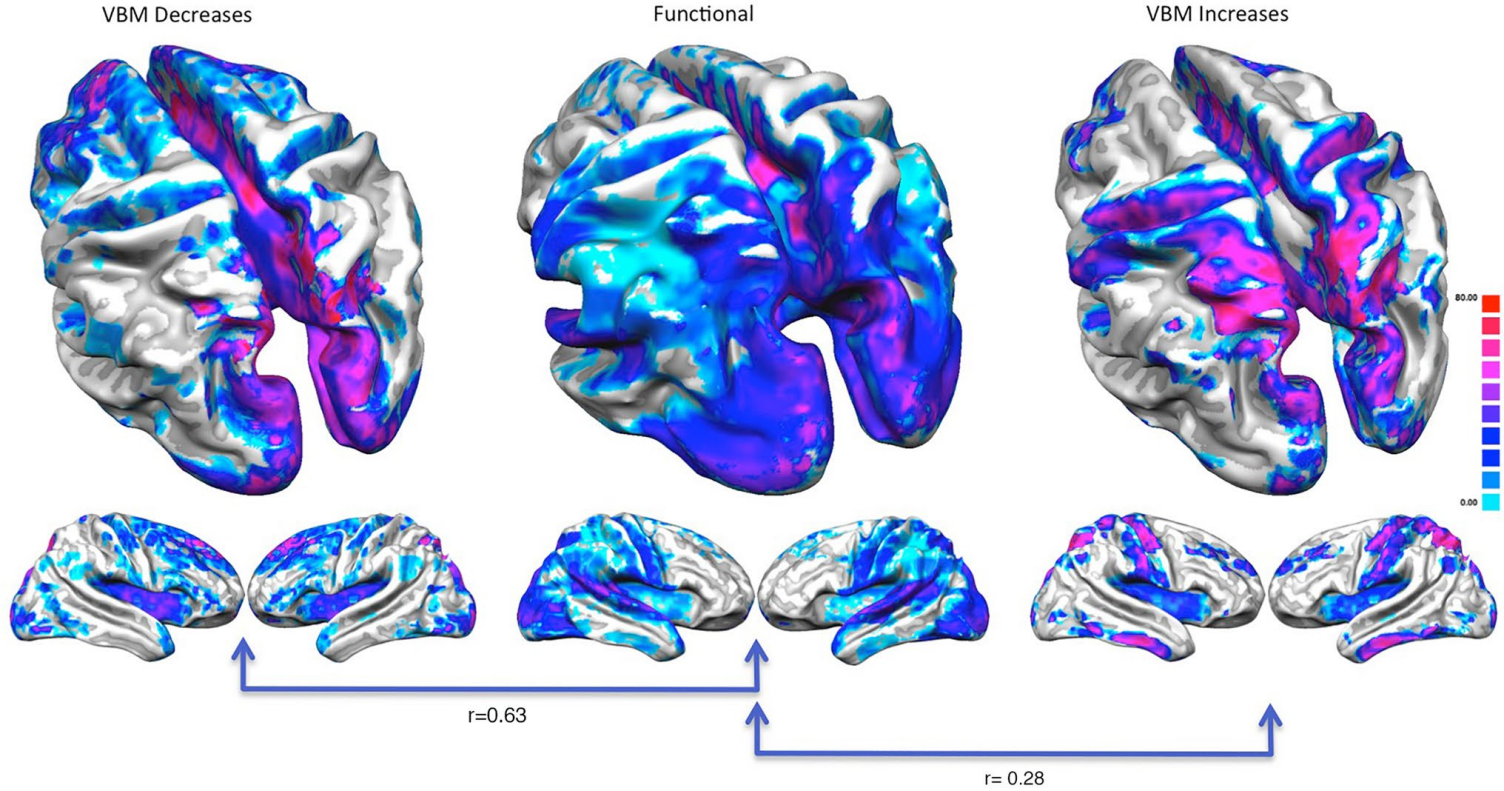
Comparison between the pathological homotopic anatomical co-alterations (PHACs) related to gray matter increases (right panel) and gray matter decreases (left panel) and the meta-analytic homotopic connectivity (MHC) (middle panel). Colors from blue to red indicate higher PHAC values. The κ values were multiplied by 100

**Fig. 4:**
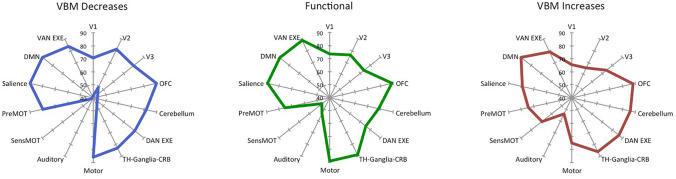
A large-scale network-based decomposition of the pathological homotopic anatomical co-alteration (PHAC) and of the meta-analytic homotopic connectivity (MHC). *V1*, *V2*, *V3* visual network 1, 2 and 3; *OFC* orbitofrontal cortex; *DAN EXE*, *VAN EXE* dorsal attentional/executive network, ventral attentional/executive network; *TH-Ganglia-CRB* thalamus, basal ganglia and cerebellum; *SensMOT* sensorimotor network; *PreMOT* premotor cortex; *DMN* default mode network. The mean κ value for each network was multiplied by 100

The increase/decrease PHACs are both similar to the MHC (Fig. 3). In fact, the co-alteration values of each couple of homotopic regions were correlated with those of the homotopic co-activation *r* = 0.63 (*p* < 0.01) for the decrease map and *r* = 0.28 (*p* < 0.01) for the increase map. This result is also clearly shown by Fig. 4, which illustrates a large-scale network-based decomposition of the results for MHC and both increase and decrease PHACs. The similarity between the functional and the decrease PHAC graphs is particularly evident, but also the morphometric increases reflect the homotopic functional connectivity in many networks. Some of the higher peaks of the decrease PHAC map (see the left panel of Fig. 4) are found in higher-order networks, such as the default mode network and the salience network, but also in primary cortices, such as the motor network. The increase PHAC (see the right panel of Fig. 4) presents a similar distribution, with lower values for all the networks, save for the default mode network. The middle panel of Fig. 4 shows the network-based decomposition of the MHC. As shown by the correlation analysis, this pattern is fairly similar to those exhibited by PHACs, especially by the decrease one. The PHAC and MHC maps obtained using the AAL atlas are quite similar to those obtained with the Talairach atlas (Supplementary Figure S2). The region-wise correlation between the PHAC and MHC analyses obtained with the AAL atlas are *r* = 0.76 (*p* < 0.01) for the decreases and *r* = 0.51 (*p* < 0.01) for the increases.

### The directional pathological homotopic anatomical co-alteration

**Fig. 5:**
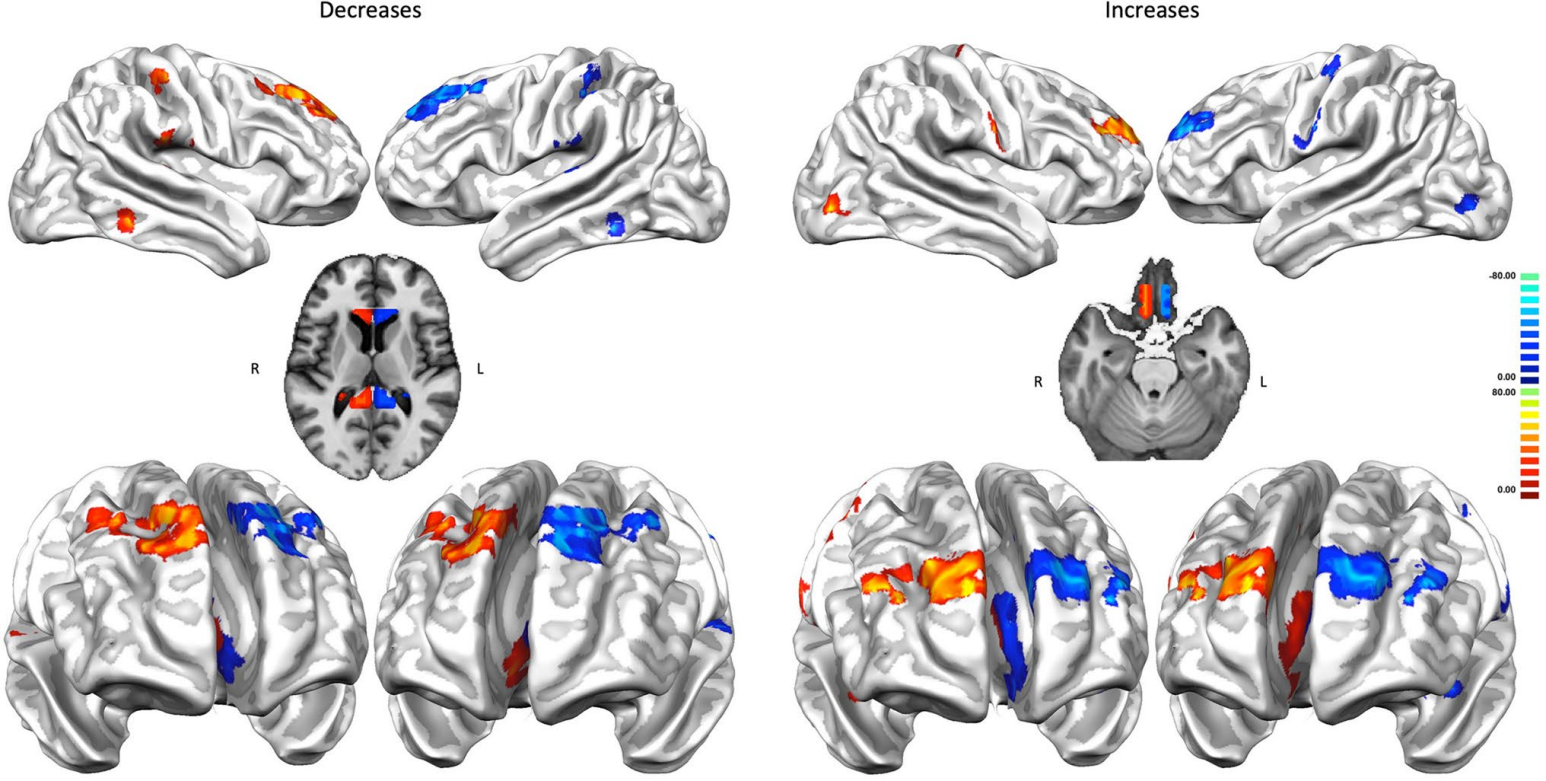
The directional pathological homotopic anatomical co-alteration (dPHAC). The left panel shows the decrease-related dPHAC, while the right panel shows the increase-related dPHAC. Colors from red to yellow indicate increased positive unbalances (directionality proceeds from positive to negative areas). Colors from dark blue to light blue indicate increased negative unbalances. The *τ* values were multiplied by 100

**Fig. 6:**
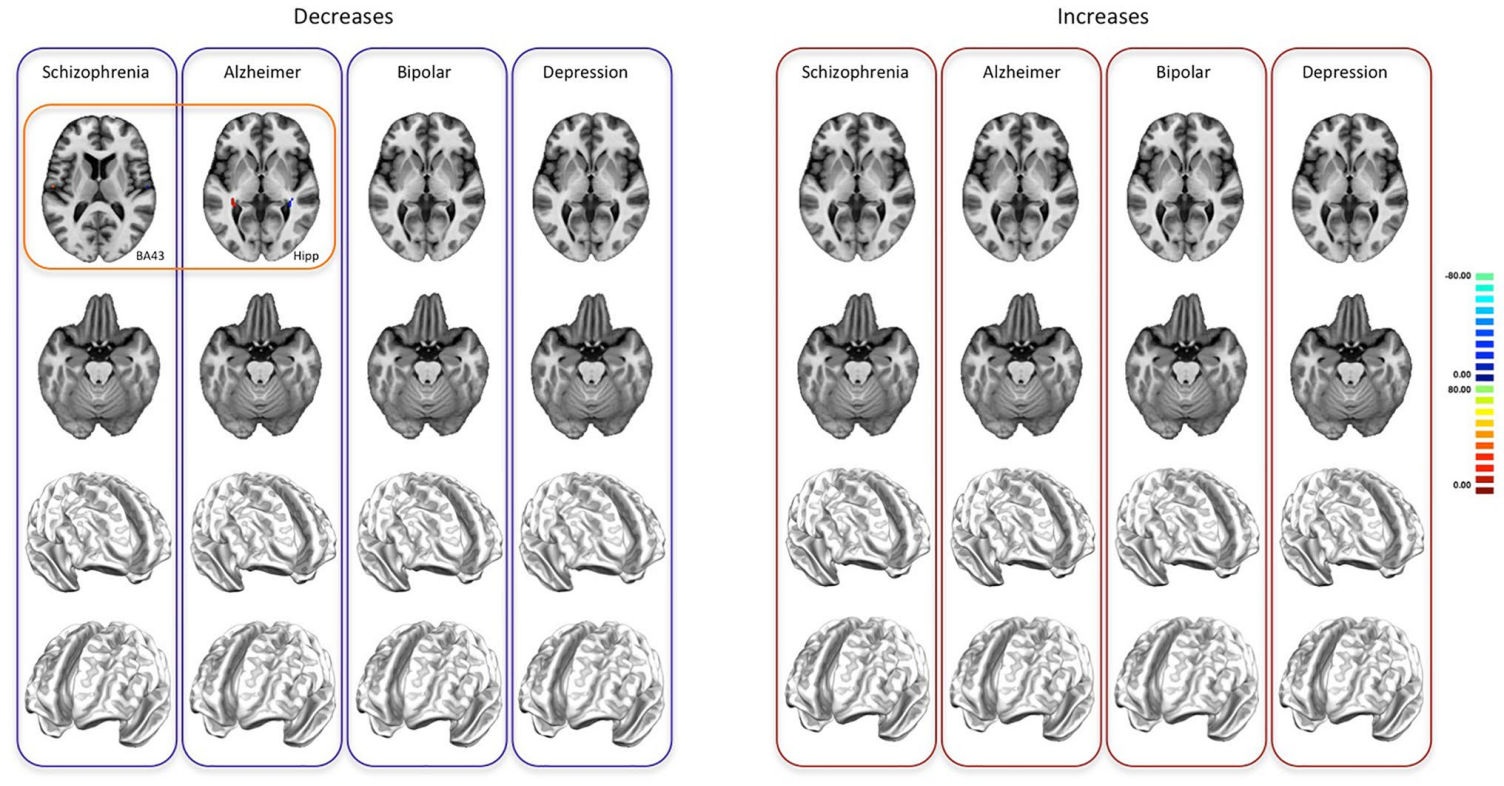
The directional pathological homotopic anatomical co-alteration (dPHAC) of the four most represented brain diseases in BrainMap [Alzheimer’s disease (AD), schizophrenia (SCZ), bipolar disorder (BD), depressive disorder (DD)]. The left panel shows the decrease-related dPHAC, while the right panel shows the increase-related dPHAC. Colors from red to yellow indicate increased positive unbalances (directionality proceeds from positive to negative areas). Colors from dark blue to light blue indicate increased negative unbalances. The *τ* values were multiplied by 100

The analysis of unbalances between hemispheres have revealed that in both the decrease (Fig. 5, left panel) and the increase (Fig. 5, right panel) dPHACs unbalances are all directed from the right to the left hemisphere. It is therefore more likely to find an alteration in a homologous area of the left hemisphere given an alteration in the right hemisphere than vice versa. However, not all the homologous couples show co-alterations characterized by significant unbalances in their conditional probability. These areas of unbalance are located in different portions of the inferior temporal, superior frontal and orbitofrontal gyri and sensorimotor brain areas. With regard to the four of the most represented brain disorders taken into consideration in this study, only the decreases of SCZ and of AD show statistically significant dPHACs, within BA 43 for SCZ and within the hippocampus for AD, respectively (Fig. 6).

**Fig. 7:**
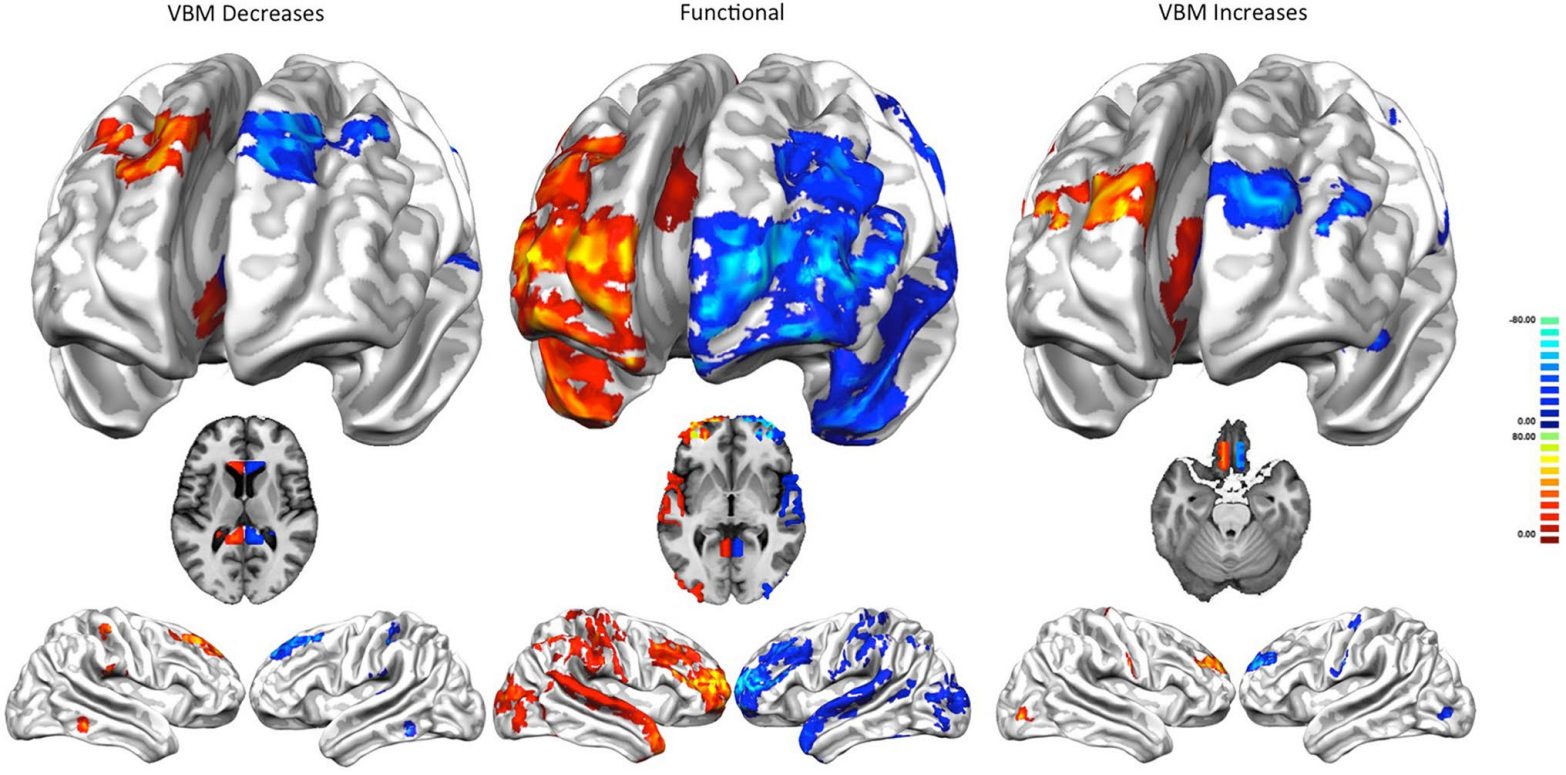
Comparison between the directional pathological homotopic anatomical co-alterations (dPHACs) related to gray matter increases (right panel) and gray matter decreases (left panel) and the directional meta-analytic homotopic connectivity dMHC (middle panel). Colors from red to yellow indicate increased positive unbalances (directionality proceeds from positive to negative areas). Colors from dark blue to light blue indicate increased negative unbalances. The *τ* values were multiplied by 100

The comparison between the increase (Fig. 7, right panel) and decrease (Fig. 7, left panel) dPHACs and the dMHC (Fig. 7, middle panel) shows also unbalances from right to left; in this case, however, the involved sites, located in superior temporal, occipital, sensorimotor, lower, middle and superior prefrontal areas, are different.

**Fig. 8:**
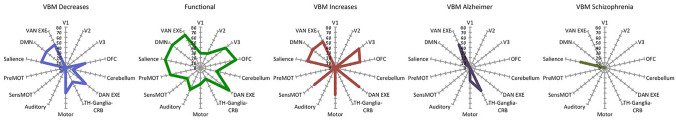
A large-scale network-based decomposition of the directional pathological homotopic anatomical co-alteration (dPHAC). *V1*, *V2*, *V3* visual network 1, 2 and 3; *OFC* orbitofrontal cortex; *DAN EXE*, *VAN EXE* dorsal attentional/executive network, ventral attentional/executive network; *TH-Ganglia-CRB* thalamus, basal ganglia and cerebellum; *SensMOT* sensorimotor network; *PreMOT* premotor cortex; *DMN* default mode network. The mean *τ* value for each network was multiplied by 100

The large-scale network-based decompositions of both the dPHACs (increase- and decrease-related) and dMHC show interesting results. While the functional dMHC presents a pattern that is relatively similar to the dPHACs, albeit with great unbalances within the integrative networks, the disease-specific dPHACs present a rather different pattern (Fig. 8). In particular, AD shows significant values in the ventral attentional network and basal ganglia, whereas SCZ in the salience network.

The fact that all analyses of dPHAC show unbalances from the right to the left hemisphere suggests a sort of right hemisphere influence over the left one. However, it should be observed that the threshold of these analyses seems to be more affected by the numerosity of the sample, as samples with fewer data (for instance, the ones related to specific brain disorders) tend to show a very limited number of significant sites.

**Fig. 9:**
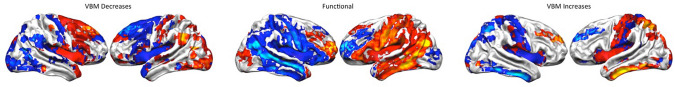
Results of the unthresholded directional pathological homotopic anatomical co-alterations (dPHACs) related to gray matter increases (right) and gray matter decreases (left) and the directional meta-analytic homotopic connectivity (dMHC) (middle). Colors from red to yellow indicate increased positive unbalances (directionality proceeds from positive to negative areas). Colors from dark blue to light blue indicate increased negative unbalances. The *τ* values were multiplied by 100

To explore this possibility, we have visualized the functional and unthresholded dPHACs (Fig. 9); we can therefore roughly estimate what could possibly happen with a larger set of data. In this case, the picture is more complex, as directionalities of unbalances are not only found from right to left.

### Bias estimation

**Fig. 10:**
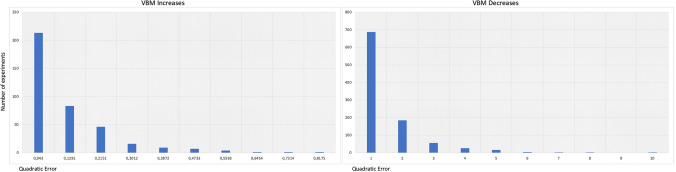
Results of the quadratic error estimation. The right panel shows the histogram related to the decreases; the left panel shows that histogram related to the increases. Most experiments present a minimal quadratic error. The quadratic error values were multiplied by 100

With regard to increases, $E_{k}$ ranges from 0 to 0.9. With regard to decreases, the range is wider, extending from 0 to 5. This suggests that data of increases present a lower error variability, while data of decreases present a higher error variability. However, the most evident and important aspect in both conditions is that most experiments present a minimal $E_{k}$. More specifically, 88% of increases and 70.1% of increases have an $E_{k}$ between 0 and 0.5, which indicates that experiments largely converge to the total ALE map (Fig. 10).

## Discussion

This study addresses three fundamental issues about the relationship between anatomical homotopic alterations. We have found that: (1) the relation between homologous areas within hemispheres is strong not only in the functional patterns of the brain at rest (Raemaekers et al. 2018), but also with regard to the anatomical alterations of the pathological brain; (2) similarly to the patterns of distribution of GM alterations across the brain (Cauda et al. 2018b), anatomical alterations within homologous brain areas show a pattern that is rather similar to the pattern of brain connectivity (in our case the meta-analytic one); (3) an unbalance in the conditional probability of directionality occurs among neuropathologically altered homologous areas, that is, given a GM alteration in a certain area of the right hemisphere, there is a greater probability to find a GM alteration in the homologous area of the left hemisphere than vice versa.

### Relationship among pathological alterations of homologous brain areas

Our results show that a strong statistical relation (i.e., co-alteration) occurs between anatomical alterations in homologous brain areas (i.e., PHAC). This is the case both for GM decreases and GM increases. Notably, certain areas (i.e., insula, medial cingulate cortex, basal ganglia, and occipital regions) exhibit high PHAC values both in GM increases and GM decreases. GM increases have higher values in sensorimotor, somatosensory and superior occipital areas, whereas GM decreases have higher values in parasagittal medial and prefrontal areas. The decomposition based on large-scale networks (Biswal 2012) reveals that both GM increases and GM decreases have higher PHAC values in associative and integrative areas and lower PHAC values in primary sensorimotor areas (Mesulam 1998). The increase PHAC (see the right panel of Fig. 4) presents lower values for many networks; however, the default mode network remains the most affected. Thus, although homotopic connectivity is known to be generally stronger in primary areas than in associative regions (Stark et al. 2008), mean PHAC values of higher-order networks, such as those of the default mode network, suggest that regions with integrative functions seem particularly affected by morphometric increases and decreases, while primary visual and auditory cortices are relatively spared. Intriguingly, with regard to the decrease PHAC the higher peaks of the network-based decomposition are, from the point of view of their functional role, the most integrative ones (see the left panel of Fig. 4); whereas the lower peaks appear to be more related to sensory functions (primary visual, auditory, and sensorimotor). To our best knowledge, this finding has never been reported before.

It is interesting to observe that the cerebellum shows a high homotopic co-alteration both in the decrease and increase map. The cerebellum is known to be an extensively connected area to the cortex; in fact, it plays an important role in learning and motor control in synergy with other cortical areas (Fine et al. 2002; Ullman 2004). Regarding functional connectivity, the cerebellum is also one of the areas with the 5% most connected voxels (Cole et al. 2010). However, any interpretation about these results have to be taken cautiously, as they might be biased by the fact that such region was likely to be outside the field-of-view of most of the MRI scans.

The discussion of these results in light of the current literature does not come without difficulties, as the PHAC is a parameter measuring a phenomenon that has never been studied before, that is, the statistical relationship among anatomical alterations of homologous brain areas. The MHC, which measures the functional connectivity between homologous areas rather than an anatomical co-alteration, is not directly related to the PHAC. Still, we expect that an association might occur between pathological anatomical co-alteration and brain connectivity, as already being shown in other studies (Cauda et al. 2012, 2017, 2018a; Crossley et al. 2014, 2016; Fornito et al. 2015; Manuello et al. 2018; Menon 2013; Raj et al. 2012; Saxena and Caroni 2011; Seeley et al. 2009; Tatu et al. 2018; Yates 2012; Zhou et al. 2012).

A measuring technique that might offer similar results to PHAC is the source-based morphometry (SBM). Like VBM, SBM is not based on a priori definition of regions of interest (ROIs) and allows an automated, user-independent study of brain structure. Differently from VBM, however, SBM uses the independent component analysis to extract spatially independent patterns occurring in structural images. In other words, VBM has a localizationist approach, as it indicates only if a voxel or a region is altered; as a result, VBM is unable to give information about the patterns of co-alteration. On the contrary, SBM takes into consideration interrelationships between voxels to pinpoint naturally grouped patterns of structural variation among populations, which can be thought of as co-alteration in the case of pathological populations (Gupta et al. 2019; Li et al. 2019). Unfortunately, this technique has been used to investigate solely specific brain disorders, whereas transdiagnostic SBM studies have not as yet been carried out.

PHAC analyses about the four most represented brain disorders in BrainMap (SCZ, AD, BD, DD) show rather different results for each disorder as well as for GM increases and GM decreases. With regard to SCZ, the PHAC obtained with GM decreases presents high values in insular, anterior and medial cingulate, medial prefrontal, postcentral, superior temporal, caudate, amygdalar and hippocampal/parahippocampal regions. With regard to GM increases, significant PHAC values only occur in the globus pallidus. These PHAC results are congruent with those found by Gupta et al. (2015) in a recent meta-analysis. In particular, the component 1 found by these authors nicely mirrors our PHAC pattern obtained from decreased data, save for the subcortical involvement, which is partly included in component 8. Although with some differences, other SBM studies have shown similar findings (Kasparek et al. 2010; Kubera et al. 2014; Xu et al. 2009a, b).

With regard to AD, the PHAC derived from GM decreases presents significant values in posterior parietal, globus pallidus and hippocampal/parahippocampal regions. As to the GM increases, only the amygdala shows relevant values. To our best knowledge, the only available SBM study about AD (Anderkova et al. 2015) confirms all the results of our PHAC analysis, save for the amygdala, which appears to be included in the decreased areas.

In case of BD, the PHAC analysis can provide significant results only with regard to GM decreases; high values have been found, especially within the insulae, the cingulate cortex and the caudate nucleus. It is worth noting that DD, which can show symptomatic analogies with BD, presents a completely different PHAC pattern (also in this case significant results are only obtained from GM decreases) exclusively formed by subcortical, amygdalar and hippocampal/parahippocampal areas. Although so far there are no SBM studies about BD, there is one about major depressive disorder (Wolf et al. 2016). This study shows rather different results compared to ours, albeit a co-alteration network (called by the authors “medial temporal lobe network”) encompasses hippocampal/parahippocampal areas.

Generally speaking, differently from the analysis of anatomical alterations, where the distinction between brain disorders is not always straightforward because of the great overlap of their alterations (Baker et al. 2014; Buckholtz and Meyer-Lindenberg 2012; Cauda et al. 2017, 2018a, 2019; Douaud et al. 2014; Ellison-Wright and Bullmore 2010; Etkin and Wager 2007; Fornito et al. 2015; Goodkind et al. 2015; Hamilton et al. 2012; Iturria-Medina and Evans 2015; Jagust 2013; McTeague et al. 2016; Menon 2013; Raj et al. 2012; Saxena and Caroni 2011; Sprooten et al. 2017; Zhou et al. 2012), the PHAC analysis allows to see subtler differences and, consequently, may discriminate better among diseases. It is worth noting that PHACs associated with specific disorders depend on the existence of morphometric abnormalities. Thus, it is particularly relevant that the PHACs seem to differentiate for each pathology better than the simple localization of the alterations. According to us, this means that the feature that better characterizes a disease is not the simple presence or absence of an anatomical abnormality in a given region, but the way in which different areas show independent or correlated modifications, which translates into the disease-related profile of homotopic co-alterations. Although the calculation of the Patel’s κ and *τ* do not take into account the probability of having both area A and area B not altered, the PHAC can discriminate between two possible cases: that one hemisphere is altered when the other is not, or that they are altered together. We explain such presence or absence of associated abnormalities as the presence or absence of a homotopic diffusion of alterations, and, using the Patel’s *τ*, we are able to estimate the directionality of a pathologic influence. Since our data are not longitudinal, our interpretation might be legitimately questioned. However, this point does not affect the finding that the PHAC analysis is able to discriminate particularly well among disease-related co-alteration profiles.

### Similarities between pathological homologous areas and meta-analytic connectivity among homologous brain areas

The second issue that this study aims to address concerns the understanding of how much homotopic connectivity patterns could influence the PHAC pattern. We have found that this influence appears to be significant: indeed, 56% and 36% of the variance concerning the decrease-related and increase-related patterns can be accounted for by the meta-analytic VMCH pattern. In other words, the pattern of statistical dependence between anatomical alterations affecting homologous areas is very similar to the functional connectivity pattern of their same homologous areas. This finding accords well with the results of the studies that compare the distribution of brain alterations with brain connectivity profiles, thus demonstrating a strict relationship between co-alteration distribution patterns and brain connectivity (Cauda et al. 2012, 2017, 2018a, b; Crossley et al. 2014, 2016; Manuello et al. 2018; Menon 2013; Raj et al. 2012; Saxena and Caroni 2011; Seeley et al. 2009; Tatu et al. 2018; Yates 2012; Zhou et al. 2012). This phenomenon can be accounted for by the fact that the mechanisms underlying the spread of neuronal alterations are likely to follow both anatomical and functional connectivity pathways (Cauda et al. 2018b)—for reviews about this topic see Fornito et al. (2015) and Iturria-Medina and Evans (2015). Given that homologous areas express higher levels of functional connectivity between each other, it is not surprising that this strong functional relationship is also mirrored in their pathological anatomic co-alteration.

### Unbalances in the directionality of the conditional probability among pathological homologous areas

The analysis of the unbalance in the conditional probability between homologous areas (i.e., dPHAC) shows in several areas a right to left hemisphere prevalence in the statistical dependence of anatomical alterations. In other words, it is more likely for an area in the left hemisphere to be altered when its homologue in the right hemisphere is also altered than vice versa. Intriguingly, both for GM increases and for GM decreases the most significant areas are located in the dorsomedial prefrontal and cingulate cortices. In particular, with regard to GM decreases, sites are located in the posterior prefrontal and in the rostral cingulate areas; whereas with regard to GM increases, in rostral prefrontal and posterior cingulate areas. In both cases, however, minor alterations are present also in postcentral areas: inferior temporal ones for GM decreases and occipital ones for GM increases, respectively. As regards to each brain disorder, only two areas of unbalance have been found: BA 43 for SCZ and the hippocampus for AD. This result is probably due to the limited number of experiments for every disease taken into consideration in this study.

It should be observed that evidence of directionality from the right hemisphere to the left might be related to the numerosity of the sample. In fact, the methodology applied here (i.e., Patel’s κ and *τ*) is influenced by the numerousness of the data (Cauda et al. 2018a). In particular, the Patel’s *τ* is calculated by employing two statistical thresholds (one for the Patel’s κ and another for the Patel’ *τ* itself) and, therefore, tends to reach significant values only with numerous data samples. For exploratory purposes, we have showed the maps of the unthresholded Patel’s *τ*. These maps present both the directionalities (from right to left as well as from left to right). For instance, within the map of the dMHC we observe a fairly dominant, albeit not yet significant, directionality from the left motor and linguistic areas to their right homologues, which is consistent with the current scientific literature about the functions of those areas in a mixed population. In light of this, it would be extremely interesting to see in future studies how sensorimotor and linguistic areas of the left hemisphere may influence their right homologues. Given the high sensitivity of the dPHAC analysis to the numerousness of the sample, some unbalances from the left to the right hemisphere may be under the statistical threshold; they could nonetheless be detected by analyzing wider or more homogeneous data sets. In any case, it is apparent that in both data sets (functional and VBM) unbalances from right to left are more intense and constant than vice versa.

Overall, our findings are particularly relevant in that they shed light in a field of research (the distribution of GM alterations between hemispheres) which at present has never been investigated. The influence of the right hemisphere on the left hemisphere within the PHAC is in accordance with several studies about humans and animals that provide evidence for this influence in a variety of contexts (both normal and pathological), ranging from active tasks performances to resting state functional and structural connectivity.

### Active tasks

A right hemisphere dominance has been repeatedly found for several tasks. For instance, it has been found for the vestibular processing (Arshad et al. 2013; Dieterich et al. 2003), spatial processing (Kinsbourne 1977) and attention (Duecker et al. 2013), bimanual grasp (Le and Niemeier 2013), spatial selective attention and target detection (Shulman et al. 2010), visual remapping (Pisella et al. 2011), as well as statistical learning (Roser et al. 2011).

### Structural connectivity

With respect to structural connectivity, unbalances in favor of the right hemisphere have been frequently found. For instance, within the corpus callosum (CC) it has been highlighted a relatively greater proportion of homotopic than heterotopic pathways towards the right hemisphere (Jarbo et al. 2012). Moreover, a right hemisphere dominance for visuospatial tasks has been associated with an anatomically larger right parieto-frontal network (Thiebaut de Schotten et al. 2011), as well as with asymmetric interhemispheric parietal connections, which can exert a greater degree of inhibition from right to left homologous areas (Koch et al. 2011). Accordingly, another study (Iturria-Medina et al. 2011) has showed in both human and non-human primate brains that the right, but not the left, posterior parietal cortex can strongly inhibit the activity of the contralateral homologous area by a short-latency connection. Intriguingly, right versus left asymmetries have been further supported by anatomical evidence in humans showing that the superior longitudinal fasciculus, which connects frontal and parietal cortices, has a right hemisphere dominance in that the volume of white matter tracts of the right fasciculus correlates positively with the detection of targets in the left compared with the right visual hemifield (Thiebaut de Schotten et al. 2011).

Overall, rightward asymmetries in the brain interconnectivity have been found both in humans and in non-human primates. These findings point out that the right posterior parietal cortex is able to inhibit the activation of the contralateral parieto-frontal connection more strongly than the left posterior parietal cortex. This effect is thought to be mediated by a transcallosal pathway located in the posterior portion of the CC.

### Functional connectivity

Studies regarding the patterns of functional connectivity across hemispheres show that the mean connectivity during resting state is more than 95% symmetric (Raemaekers et al. 2018), which implies that at best the functional asymmetries are modest. Functional differences in favor of a right hemisphere dominance have been found in a study by Medvedev (2014), which revealed significantly higher connectivity in the right hemisphere in the majority of right-handed individuals and in the two left-handed individuals that participated in the experiment. Gotts et al. (2013) have found that areas of the right hemisphere reveal a more bilateral functional connectivity than areas of the left hemisphere, which interact more strongly with themselves. However, these asymmetries were less clearly highlighted by other studies (Joliot et al. 2016; Wang et al. 2014).

### Effective connectivity

With respect to effective connectivity, proof of a right hemisphere dominance has been obtained. Medvedev (2014) carried out a Granger causality analysis across the hemispheres on resting state data, which showed an influence of the right hemisphere on the left one. Another study of effective connectivity by Dietz et al. (2014) has found that the right hemipshere is dominant on the left one in audiospatial perception. These findings are in accordance with the right hemisphere dominance model proposed by Heilman and Van Den Abell (1979) and Mesulam (1981). These results are also in line with the observation that, during visuospatial attention tasks, the right parietal cortices exert an inhibitory function over the left ones (Koch et al. 2011).

### Clinical studies

The existence and relevance of an interindividual variability of brain asymmetry that is related to behavioral, physiological or personological features have been repeatedly confirmed. For instance, altered asymmetries were found to be related to a reduction in functional connectivity as well as to clinical manifestations such as auditory hallucinations (Oertel-Knochel er al. 2013). Moreover, SCZ patients were found to present a volumetric rightward asymmetry of amygdala and hippocampus (Okada et al. 2016; Qiu et al. 2009), suggesting the possibility of an anomalous lateralization of neuronal patterns in SCZ. Further abnormalities of GM hemispheric asymmetries, possibly genetically determined (Crow 1998), have been found in patients with SCZ (Bilder et al. 1994), and it is also believed that an incomplete lateralization contribute to SCZ (Frith 2005; Stephane et al. 2001). Finally, depression has been associated with an unbalanced interhemispherical activity (Flor-Henry et al. 2004; Henriques and Davidson 1991; Nielsen et al. 2013). These examples do not provide evidence of a strict disease-specificity for brain asymmetries, as an identification of brain pathology based on anatomical data is rather challenging (Cauda et al. 2019). However, these studies show that an unbalance between hemispheres can be a feature of many diseases; therefore, they provide a context for our findings, which in turn appear to accord well with the existing literature about the interhemispheric interaction in brain pathology.

Furthermore, two recent studies from our group also provide evidence for a hemispheric dominance in pathology. First, the hubs of long-distance co-alteration of a transdiagnostic network of anatomical decreases were particularly located in certain regions of the left hemisphere, such as the dorsolateral prefrontal cortex and the sensorimotor cortex, while those of the network of increases were found in the homotopic areas of the opposite hemisphere (Cauda et al. 2020). Secondly, calculating a network of interdependence between VBM decreases and increases of psychiatric disorders, we observed that its hubs were especially located in the left hemisphere, thus suggesting a left hemispheric dominance on the mechanisms of anatomical compensation (Mancuso et al. 2020).

### Animal studies

Animal studies provide support for the asymmetries found in humans. For instance, baboons present a right hemisphere dominance for emotion processing (Wallez and Vauclair 2011). Furthermore, the study by Iturria-Medina et al. (2011) has pointed out both in humans and in non-human primates the same short-latency transcallosal inhibitory mechanism exerted by the right parietal cortex in controlling the contralateral homologous area.

### Anatomical studies

A rightward asymmetry has been commonly reported for the hippocampus and amygdala (Kallai et al. 2005; Pedraza et al. 2004; Wang et al. 2001). Greater volume asymmetries in the right hemisphere than in the left have been found in the thalamus, caudate nucleus, putamen, and nucleus accumbens (Deicken et al. 2002; Gunning-Dixon et al. 1998; Qiu et al. 2009; Wyciszkiewicz and Pawlak 2014; Yamashita et al. 2011). In patients with SCZ an abnormal pattern in the ratio between the left and right lateral ventricular volumes (in normal individuals there seems to be an asymmetry in favor of the left lateral ventricle) has been highly correlated with thought disorder (Shenton et al. 1991). Also, asymmetry of the *planum temporale* and the Sylvian fissure has been found in patients with SCZ (Sommer et al. 2001).

Notably, Tanaka et al. (2012) have identified a trend for a greater rightward asymmetry of cortical GM volume with regard to all brain regions. Overall, the right hemisphere has a larger blood supply than the left one, and there is a higher mortality in cases of similar but right-sided hemispheric lesions. A study by Arshad et al. (2015) has found that the individuals with greater right hemisphere dominance have at the baseline a less excitable primary visual cortex and are able to exert a greater degree of top-down modulation over the low-level brain mechanisms, such as the brainstem-mediated vestibular-ocular reflex.

### The relationship between homotopic connectivity and pathological co-alteration

The present study focuses on the pathologic connectivity between homotopic areas. However, brain diseases are known to also produce intrahemispheric anatomical co-alteration. The interhemispheric effect of brain pathology on connectivity has been reported in a study of SCZ, autism spectrum disorder and depression (Zhang et al. 2015). The authors observed that changes in functional connectivity between two regions could largely be accounted for by functional connectivity of these regions with their interhemispheric homologue. Thus, homotopic connectivity might play a role in the development of brain alterations. Furthermore, it is well known that commissures mostly connect homotopic regions (Raybaud 2010), and functional homotopic connectivity shows to be strong throughout the brain (Stark et al. 2008). Homotopic connectivity might constitute a significant route of interhemispheric spread of toxic agents. Indeed, many regions with high PHAC values, such as the insula, anterior cingulate cortex, and thalamus are also known to be areas that are frequently co-altered by brain diseases (Cauda et al. 2018a; Crossley et al. 2014; Goodkind et al. 2015). In virtue of their centrality in functional connectivity, they could act as nodes favoring the alterations’ spread between intrahemispheric and interhemispheric connected areas. Mechanisms of transneuronal spread have been proposed in neurodegenerative diseases (Goedert et al. 2017; Guest et al. 2011; Raj et al. 2012; Seeley et al. 2009; Zhou et al. 2012), as well as in psychiatric disorders (Atkin et al. 2012; Guest et al. 2011; Zhu et al. 2017). This could explain why pathological co-alterations seem to develop in a network-like fashion. In this view, homotopic connectivity could make it possible for alterations to propagate to the contralateral hemisphere, leading to a pathological co-alteration of GM decreases, or might recruit areas that are homologous to those already altered in an attempt of functional compensation, leading to a pathological co-alteration of GM increases.

### Homotopic morphometric alterations and interhemispheric pathways

If pathological gray matter changes follow connectivity routes, it is plausible to assume that the main paths to be followed by homotopic alterations are the CC and the other telencephalic commissures (anterior commissure and hippocampal commissure), since commissural, and in particular callosal, connections are more often homotopic than heterotopic (Hedreen and Yin 1981; Jarbo et al. 2012; Raybaud 2010).

With regard to the CC, it has been long debated if its function is mainly excitatory or inhibitory, that is, if its connections produce a mutual exchange of information or a mirrored inhibition that underlies functional and anatomical asymmetries (Bloom and Hynd 2005; van der Knaap and van der Ham 2011). It is more likely that both transfer of information and mutual inhibition are carried out by the CC, depending on the task and cognitive load (Bloom and Hynd 2005). According to this view, we can hypothesize that both pathological GM increases and/or GM decreases might occur in the same area, depending on what interhemispheric mechanism the CC is involved in. This is not to say that GM increases are solely the effect of a greater pathological excitation via the CC, whereas GM decreases are solely caused by a greater pathological inhibition via the CC. Other processes mediated by the CC could play a role, such as factors of excitotoxicity for GM decreases and compensatory mechanisms for GM increases. More precisely, we are hypothesizing that, being the CC a complex structure that sustains different forms of interhemispheric interactions, its complexity might be the key to explain the large overlaps between the increase and decrease PHAC maps, and their similarity to that of the functional homotopic connectivity.

Although the CC is the major interhemispheric commissure, other structures could be of some importance. For instance, the anterior commissure is known to connect portions of temporal, occipital and frontal lobes (Di Virgilio et al. 1999). Furthermore, the observation of a split-brain patient with complete telencephalic commissurotomy and relative maintained visual transfer (Eviatar and Zaidel 1994) and intact interhemispheric functional connectivity (Uddin et al. 2008), seems to underline the capability of subcortical commissures (habenular, tectal, collicular and posterior commissures) (Aralasmak et al. 2006) of supporting communication between the hemispheres, thus suggesting that also these commissures might play a role in pathoconnectivity.

### Limitations and future directions

Some methodological considerations are needed, as they are important for the interpretation of our results.

(1) A possible confounding factor is that, to create a symmetrical atlas, we overlapped the right hemisphere on the left. This choice was a methodological constraint, as the use of an asymmetrical atlas would have been much more problematic. However, we believe that this choice could contribute to decrease the PHAC values and not to increase them, thus ruling out the inflation of false positives.

(2) The ROIs were defined thanks to an anatomical atlas; therefore, they might fail to take into account possible more detailed subdivisions in heterogeneous regions (Cieslik et al. 2016; Genon et al. 2017). On the other hand, the ROIs division made possible to achieve more powerful statistical scale to ascertain an asymmetry. In theory, although smaller ROIs would allow a more detailed investigation, this choice would have increased the number of errors in the definition of the ROIs, thus reducing statistical power of the study.

(3) Since we used an atlas with areas of different size, smaller areas are more likely to be found altered; in fact, they reach more easily the 20% threshold of altered voxels (see “Materials and methods”). However, we previously showed that the bias introduced by the size of the volumes is not significant (Mancuso et al. 2019). Instead, the alternative way, which builds a parcellation with same-sized volumes (Fornito et al. 2010; Zalesky et al. 2010), produces more biased results those obtained with the Talairach atlas (Mancuso et al. 2019).

(4) The VBM studies included did not specifically aim at detecting asymmetries and, consequently, did not apply any image processing related to this issue—e.g., the use of a symmetric template that would have ensured a precise interhemispheric correspondence.

(5) The effect of handedness might be a confounding factor of our analyses, as our database included both right- and left-handed participants.

(6) Our method is basically a group-level structural covariance, in which each BrainMap experiment represents a group. On one hand, a structural covariance study should reassure the reader about the feasibility of finding a statistical association between the morphology of brain regions. On the other hand, working on groups rather than on single-subjects might raise concerns about the plausibility of our results. One may argue that, within a given group, some subjects could have alterations only in the right hemisphere and other subjects only in the left one, so that the second level analysis would represent poorly this inter-individual variability as foci of significant effect on both the hemispheres. Hypothetically, such extreme case could indeed affect the results.

Of note, it can be shown that even considering the most permissive threshold that is currently used: *p* ≤ 0.05 it is not possible that a VBM pattern showing a bilateral co-alteration might be the spurious result of a pathological group in which subjects have only homolateral lesions, because the effect would not be enough to go beyond the threshold of the statistical significance (see the Supplementary Materials for a detailed explanation).

Moreover, Patel’s calculus can assess the three following possibilities: (i) left area A and right area B are co-altered in the same experiment (and, therefore, in the same group); (ii) left area A is altered but right area B is not altered in the same experiment; (iii) left area A is not altered but right area B is altered in the same experiment. Patel’s κ value increases when occurrences of case (i) increase. In other words, Patel’s κ value is higher when the alteration is present in both homotopic areas in the same experiment or, with regard to single-subject data, in the same individual, and not when an alteration is present in one hemisphere with regard to an experiment/patient and an alteration is present in the other hemisphere with regard to another experiment/patient. Undoubtedly, within an experimental group it is possible to find patients with different alteration patterns, which could lead to a group analysis masking individual variability.

Therefore, the reliability of our technique depends on the reliability of the data. Within the field of neuroimaging, some authors (Finn et al. 2015) have pointed out that group-level analyses might hide meaningful inter-individual differences; however, it is unlikely that everything done as regular second level analysis is to be disregarded. Still, it could be possible that a large amount of VBM studies and activation literature should be erroneous in that they do not represent what actually happens in the individual brain. For this reason, our findings are to be considered with caution. Further investigations are needed to test the validity of the classic group-level statistics in neuroimaging. Furthermore, Carmon et al. (2020) have highlighted the variance between the results of different structural studies and pipelines. On one hand, we might expect that a part of this variance is lost when the group-level statistics is resumed in a single coordinate, which is the only data utilized by our calculation. On the other hand, the advantage of performing a meta-analysis is exactly to overcome the methodological differences between single studies. Thus, we believe that the challenges in performing structural covariance over different sites or studies are largely mitigated by our coordinate-based meta-analytical methodology.

(7) The dPHAC is an innovative calculus, as the use of an empirical Bayesian technique to estimate the unbalance of conditional probabilities has never been applied before on VBM meta-analytical data. Results of this analysis, therefore, should be interpreted cautiously and need to be supported by future studies. Furthermore, our results are exclusively based on aggregate meta-analytical data and not on single-subject data. At present there are many databases combining a great number of single-subject data. Although we have performed plenty of comparisons with analyses conducted on single-subject data, to date some results have not been replicable. This is so because our methodology cannot analyze single-subject data. We plan therefore to create a method capable of replicating thoroughly the results presented here on single-subject data as well; if achieved, this will provide further support for our findings.

(8) Although the BrainMap database accepts only peer-reviewed articles reporting whole-brain coordinate-based (*x*, *y*, *z*) results, it may contain a limited number of neuroimaging experiments that do not have correction for multiple comparisons and that, instead, use a more liberal thresholding. Unfortunately, the automated search algorithms of BrainMap do not allow the selection of neuroimaging experiments on the basis of the statistical threshold or the correction for multiple comparisons. Because of this, it is possible that our sample include experiments with thresholds that are more liberal than those which are currently used. This could have increased the number of false positives but, given the methodology applied here, which aims to identify concordance of results across experiments, we think that a limited number of experiments with more liberal thresholds would not have biased significantly our results. This consideration is further supported by the bias estimation analysis carried out on our sample, which indicates a large converge of experiments to the total ALE map. This finding leads us to think that a possible bias due to studies with more liberal thresholds is negligible, as the number of these experiments is so limited as to not affect our results. Nonetheless, other studies are needed to provide evidence and further validation of our findings.

## Conclusion

This study focuses on three issues about the relationship between homologous areas in the pathological brain that, to our best knowledge, have never been addressed before: (1) is there a statistical relationship between the anatomical alterations of homologous areas caused by brain diseases? (2) Can the pathological co-alteration of homologous areas be influenced by brain connectivity patterns? (3) Is there a directionality in the probability of homologous areas to be co-altered?

Our analysis provides evidence that (1) not only at rest the homologous areas are functionally linked, but also in case of pathological processes they appear to be anatomically co-altered. (2) This co-alteration pattern or pathological co-alteration is very similar to the pattern of brain connectivity exhibited by the couples of homologues. Finally, (3) we have discovered that the probability to find alterations in the areas of the left hemisphere seems to be greater when their right homologous are also altered than vice versa, an intriguing asymmetry that deserves to be further investigated and explained. If confirmed by future studies, these important findings can shed further light on the dynamics of neuropathological processes and support a pivotal role of the right hemisphere in the spread and distribution of anatomical alterations caused by brain disorders.

## Supplementary Information

Below is the link to the electronic supplementary material.

Supplementary file1 (DOCX 9402 KB)
