# Supplementary material for: Interhemispheric co-alteration of brain homotopic regions
Source: Brain Struct Funct. 2021 Jun 25;226(7):2181–204. doi: 10.1007/s00429-021-02318-4 (PMC8354999; doi:10.1007/s00429-021-02318-4)
Supplement: Supplementary file 1 — Supplementary file1 (DOCX 9402 KB) [file 429_2021_2318_MOESM1_ESM.docx]

##### Interhemispheric co-alteration of brain homotopic regions

Supplementary Material

**1. Selection of Studies: literature search strategy**

Neuroimaging experiments included in the meta-analysis were retrieved from the online BrainMap database (http://www.brainmap.org/) (Fox and Lancaster 2002; Laird et al. 2005; Vanasse et al. 2018). Using a standardized taxonomy, this database contains meta-data associated with over 4000 peer-reviewed publications that report whole-brain coordinate-based (x,y,z) results in a stereotactic space. BrainMap ensures a rigorous quality control of the coding for every paper. Most meta-data information may be found in the sitemap, such as the article abstract, pubmed ID, class of subjects, software package and type of analysis. Although BrainMap focuses on experimental design being used, it does not allow the selection of experiments based on single aspect of this information. This sometimes coincides with the inclusion of neuroimaging experiments that do not have correction for multiple comparisons, but which represent a limited part of the database. We therefore considered all the experiments as potentially eligible for our analysis. To date, over 950 peer-reviewed papers using BrainMap data and software have been published.

The software application Sleuth (v.2.4) (Fox et al. 2005) has been used to search the dataset of interest and view the relevant search results in a stereotaxic brain space. The search algorithms were constructed as follows:

1. For the decreases (voxel-based morphometry dataset): [*Experiments Context is Disease] AND [Experiment Contrast is Gray Matter] AND [Experiments Observed Changes is Controls>Patients]*;
2. For the increases (voxel-based morphometry dataset): [*Experiments Context is Disease] AND [Experiment Contrast is Gray Matter] AND [Experiments Observed Changes is Patients>Controls]*;
3. For the functional data (functional dataset): [*Experiments Context is Normal Mapping] AND [Experiments Activation is Activations Only] AND [Subjects Diagnosis is Normals].*

*1.1. Assesment of eligibility of studies*

All the voxel-based morphometry (VBM) experiments identified by our standardized searches were evaluated in order to ensure that they comply with specific eligibility criteria. Studies were included if:

1. they were original studies published in a peer-reviewed English language journal;
2. they used a well-specified VBM analysis;
3. they adopted a whole-brain analysis (i.e., field of view not confined to a restricted region of interest);
4. they compared pathological sample with matched healthy control participants;
5. they reported gray matter (GM) decrease/increase changes in the pathological sample;
6. the locations of GM alterations were reported in Talairach/Tourneaux (TAL) or in Montreal Neurological Institute (MNI) stereotaxic space;
7. they were not animal models;

Transparent and complete report of data selection is shown in Figure S1 [PRISMA flow chart] (Liberati et al. 2009; Moher et al. 2009).

*1.2. Data extraction*

The software application Sleuth (v.2.4) has been used to extract the meta-data of GM alterations from each selected experiment. In order to facilitate analysis, locations from MNI stereotactic space were converted into TAL space by using Lancaster’s icbm2tal transformation (Laird et al. 2010; Lancaster et al. 2007). For more information about the description and distribution of the extracted VBM data, see Tables S1 and S2. Finally, we carried out the analysis on the functional BrainMap dataset in order to acquire the largest possible set of data, with the intention to obtain our meta-analytic homotopic connectivity map. For more information about the selected functional data, see Table S3.

**1.2 Procedure evaluation**

In order to validate our methodology, we could imagine a hypothetical extreme situation that might create spurious results. If our technique proves to be able to be not affected by artefactual co-alterations in such worst case scenario, we could conclude that our results are accurate.

The hypothetical case is the following. Let a given VBM experiment be the result of a group statistic calculated on a sample of patients, divided in two subsample 1 and 2. Subsample 1 and 2 have the same size (the two subsample split the global sample in two equal parts), and have the following characteristic: all the patients of subsample 1 have an alteration in region A, and none have an alteration in region B, while all the subjects of subsample 2 have an alteration in region B and node in region A. Since the present research is about homotopic co-alterations, region A and B can be considered as two homotopic regions in the left and right hemisphere, respectively, but the reasoning holds true also for any other couple of nodes. This hypothetical case represent a situation of real independence of alteration that might produce a spurious co-alteration, and thus possibly decrease the accuracy of our results.

The first step is just to estimate the likelihood for this case to present. Considered that the average number of subjects of our database can be approximately estimated to be of 30 subjects, let the N_sample_=30 and thus N_subsample1_=N_subsample2_=15. If the events are independent, the joint probability is expressed as follows:

$$p\left(A,B\right)=p\left(A\right)p\left(B\right)$$

Considering the case of having half subsample with an alteration in A and not in B with N_subsample1_=N_subsample2_, the probability of this condition is binomial, as:

$$p\left(m\right)=\left(\begin{matrix} n \\ m \end{matrix}\right)p^{m}q^{n-m}$$

Assuming a probability of p=0.5, the p-value is about 0.144. Thus, the probability to have an alteration in A and not in B in half subsample and the opposite in the other one results from the product of the two probabilities, that is 0.0207. The joint probability in the double dissociation case is thus about 2%. Thus, we evaluated that this case is extremely rare.

Our second step is now to calculate the probability that the VBM statistical test could present an alteration in both node A and B given the double dissociation case. Let us simulate the calculation as in FSL. First, it is necessary to use a GLM assuming continuous data (Jenkinson et al., 2012). The following MATLAB code would simulate the statistic test:

% glm simulation like in FSL

clear all

close all

n=30;

mean_diff=1;

s=1;

for i=1:5000

v1_patients1=2*mean_diff+s*randn(n/2,1);

v1_patients2=randn(n/2,1);

v1_patients=[v1_patients1; v1_patients2];

v1_controls=randn(n,1);

Y=[v1_patients; v1_controls];

dum1=[ones(n,1);zeros(n,1)];

dum2=[zeros(n,1);ones(n,1)];

X=[dum1 dum2];

beta_hat=inv(X'*X)*X'*Y;

Var_e=(Y-X*beta_hat)'*(Y-X*beta_hat)/(n-1-length(beta_hat));

c=[1;-1];

t_stat(i)=c'*beta_hat/sqrt (Var_e*c'*inv(X'*X)*c);

end

th=tinv(1-0.05/2,n-1-length(beta_hat));

mean(t_stat > th)*100

The GLM simulation results show the following: considering a threshold of 0.05 we have a probability of obtaining a false co-alteration of 78.6%, only taking a two-tailed test into account. If we use the more commonly utilized one-tailed statistic, because in VBM we consider only increase or only decrease separately, we should set the threshold to 0.025, thereby obtaining a p of about 54.6%, which is relatively low compared to what is commonly recognized as a good power, i.e. ≤ 80%. Furthermore, it is important to note that in FSL the default threshold for the VBM calculation is set to 0.01, thus we obtain a power to detect a false co-alteration about 29.3%.

In the context of meta-analyses the main purpose is to integrate the information from several experiments so that the amount of noise in the data is reduced and the chance of producing false positives is minimized. To achieve this many method are available: in our study, we used the Patel’s κ. As detailed in the article, Patel’s κ is based on the following contingency table:

| VOI *b* | VOI *a* | | | |
| --- | --- | --- | --- | --- |
|  |  | Altered | Unaltered |  |
|  | Altered | $\theta_{1}$ | $\theta_{3}$ | $\theta_{1}+\theta_{3}$ |
|  | Unaltered | $\theta_{2}$ | $\theta_{4}$ | $\theta_{2}+\theta_{4}$ |
|  |  | $\theta_{1}+\theta_{2}$ | $\theta_{3}+\theta_{4}$. | 1 |

and on the following formula:

$$\kappa=\frac{\vartheta_{1}-\left(\vartheta_{1}+\vartheta_{2}\right)\left(\vartheta_{1}+\vartheta_{3}\right)}{D-\left(max\left(\vartheta_{1}\right)-E\right)+\left(1-D\right)\left(E-min\left(\vartheta_{1}\right)\right)}$$

where

$$E=\left(\vartheta_{1}+\vartheta_{2}\right)\left(\vartheta_{1}+\vartheta_{3}\right)$$

$D=\left\{\begin{matrix} \frac{\vartheta_{1}-E}{2\left(max\left(\vartheta_{1}\right)-E\right)}+0.5,if\vartheta_{1}\geq E \\ 0.5-\frac{\vartheta_{1}-E}{2\left(E-min\left(\vartheta_{1}\right)\right)},otherwise \end{matrix}\right.$.

$$max\left(\vartheta_{1}\right)=min\left(\vartheta_{1}+\vartheta_{2},\vartheta_{1}+\vartheta_{3}\right)$$

$$min\left(\vartheta_{1}\right)=max\left(0,2\vartheta_{1}+\vartheta_{2}+\vartheta_{3}-1\right)$$

From the formula we can observe that the Patel’s κ is the difference between the probability that a and b are altered together and the expected probability that a and b are independently altered, and the denominator is a weighted normalizing constant. Thereby, it takes the possibility that a and b are altered independently into account.

We already shown that the probability of having an alteration in the right hemisphere in half of the sample of a given experiment and an alteration in the right one in the other half, would be extremely low (0.02). However, it is clear that a single false co-alteration in the database cannot influence the Patel’s κ value if all the remaining experiments are true. Thus, to really put our method in the condition of producing a false co-alteration, let us hypothesize an even more unlikely situation, that is, a situation in which all the experiments of the database are the result of a double dissociation case as that one presented above. Considering the decrease dataset whose size is N_decreases_=980, the probability of such situation would be 0.02980.

We showed that the probability that such case can be mistaken by the VBM for a case of co-alteration in the 29.3% of the time, thus we could imagine that the 29.3% of the experiments would present a false co-alteration between nodes A and B, while the remaining would correctly show an absence of co-alteration. For simplicity, let us imagine that those remaining 71% of cases would simply split equally between θ2 and θ3, that is, the probability of having an alteration in A and not B and the probability of B and not A, respectively. If we place this value in the previous table, the following situation emerges:

| VOI *b* | VOI *a* | | | |
| --- | --- | --- | --- | --- |
|  |  | Altered | Unaltered |  |
|  | Altered | 0.293 | 0.3535 | 0.6465 |
|  | Unaltered | 0.3535 | 0 | 0.3535 |
|  |  | 0.6465 | 0.3535 | 1 |

The result of the Patel’s κ would be:

$$\kappa=\frac{0.29-0.65*0.65}{1\left(0.65-0.42\right)+\left(1-1\right)\left(0.42-0.29\right)}=\frac{0.293-0.42}{0.65-0.42}=-0.55$$

However, we might want to assume that, the 29.3% of the cases in which the VBM produce a false results, the result would not be necessarily a false co-alteration, but possibly also be a false negative, in which no alteration would be detected at both the right and left nodes. Again, for the sake of simplicity, we could say that half of the mistakes would be false positives, and the other half false negatives, thus we split the p=0.293 between θ1 and θ4. The table would become:

| VOI *b* | VOI *a* | | | |
| --- | --- | --- | --- | --- |
|  |  | Altered | Unaltered |  |
|  | Altered | 0.146 | 0.3535 | 0.5 |
|  | Unaltered | 0.3535 | 0.146 | 0.5 |
|  |  | 0.5 | 0.5 | 1 |

And thus:

$$\kappa=\frac{0.15-0.5*0.5}{0.71\left(0.5-0.25\right)+\left(1-0.71\right)\left(0.25-0\right)}=\frac{0.15-0.25}{0.71*0.25+0.29*0.25}=-0.42$$

Since both of the cases would produce a strongly negative value, the calculations clearly show that our method would not detect a spurious co-alteration between the two nodes. Rather, negative values represent the case in which the observed co-alteration are less likely than the expected probability in a condition of independence between the two nodes. Thus, our method is perfectly able to discriminate true homotopic co-alterations from artefactual ones, even in the most extreme and unrealistic scenarios.

Furthermore, this situation has been proposed just for the couple of nodes A and B, but since in the Talairach Atlas used in the present paper there are approximately 1000 areas, the calculation of the homotopic co-alteration has been repeated ~500 times. Also, if we were interested in the whole co-alteration network, we would have calculated [1000*(1000-1)]/2 edges. Thus, in general, the probability that the hypothetical double dissociation case is presenting so often to be considered a problem is abysmal. That said, even if it does, we can safely conclude that it would not introduce false positives (that is, Patel’s κ with a falsely positive value) in our results.

**1.3 Subsampling reliability**

In order to test the reliability towards subsampling of the results obtained through the Patel’s k and tao indexes, we implemented a bootstrap procedure with 5,000 iterations. In each run the 50% of the VBM experiments were randomly sampled, independently for decrease and increase conditions. Based on each sub-sample, k and tao were computed between the couples of homotopic parcels that showed a significant co-alteration in the main analysis. Finally, the standard error of k and tao values across the 5,000 runs was computed for each couple. Results showed an overall robust reliability (see figure S4).

**References**

Fox PT et al. (2005) BrainMap taxonomy of experimental design: description and evaluation Human brain mapping 25:185-198 doi:10.1002/hbm.20141

Fox PT, Lancaster JL (2002) Opinion: Mapping context and content: the BrainMap model Nature reviews Neuroscience 3:319-321 doi:10.1038/nrn789

Jenkinson M, Beckmann CF, Behrens TE, Woolrich MW, Smith SM (2012) FSL NeuroImage, 62:782-90

Laird AR, Lancaster JL, Fox PT (2005) BrainMap: the social evolution of a human brain mapping database Neuroinformatics 3:65-78

Laird AR et al. (2010) Comparison of the disparity between Talairach and MNI coordinates in functional neuroimaging data: validation of the Lancaster transform NeuroImage 51:677-683 doi:10.1016/j.neuroimage.2010.02.048

Lancaster JL et al. (2007) Bias between MNI and Talairach coordinates analyzed using the ICBM-152 brain template Human brain mapping 28:1194-1205 doi:10.1002/hbm.20345

Liberati A et al. (2009) The PRISMA statement for reporting systematic reviews and meta-analyses of studies that evaluate health care interventions: explanation and elaboration Journal of clinical epidemiology 62:e1-34 doi:10.1016/j.jclinepi.2009.06.006

Moher D, Liberati A, Tetzlaff J, Altman DG (2009) Preferred reporting items for systematic reviews and meta-analyses: the PRISMA statement Journal of clinical epidemiology 62:1006-1012 doi:10.1016/j.jclinepi.2009.06.005

Vanasse TJ, Fox PM, Barron DS, Robertson M, Eickhoff SB, Lancaster JL, Fox PT (2018) BrainMap VBM: An environment for structural meta-analysis Human brain mapping 39:3308-3325 doi:10.1002/hbm.24078

Supplementary Tables and Figures

**Figure S1 [PRISMA flow chart]**. Overview of the selection strategy.

AD = Alzheimer’s disease; SCZ = schizophrenia; BD = bipolar disorder; DD = depressive disorder.

Identification

**2376 Functional Records** identified through **BrainMap**

**994** **VBM Records** identified through **BrainMap**

Screening

**2376** Records after duplicates removed

**994** Records after duplicates removed

**2376** Records screened

**994** Records screened

Eligibility

**994** Full-text articles assessed

for eligibility

Full-text articles excluded

87 not whole-brain analysis

63 not healthy controls

29 white matter analysis

22 not clear sample characteristics

Included

**2376 functional studies** included in

quantitative synthesis

**793 VBM studies** included in

quantitative synthesis

54

BD

46

DD

55

AD

114

SCZ

524

OTHERS

**Figure S2:** The maps obtained with the AAL atlas. The left panel shows the decrease-related pathological homotopic anatomical co-alteration (PHAC), while the right panel shows the increase-related PHAC. The bottom panel shows the meta-analytic connectivity. Colors from blue to red indicate higher PHAC values. The κ values were multiplied by 100.


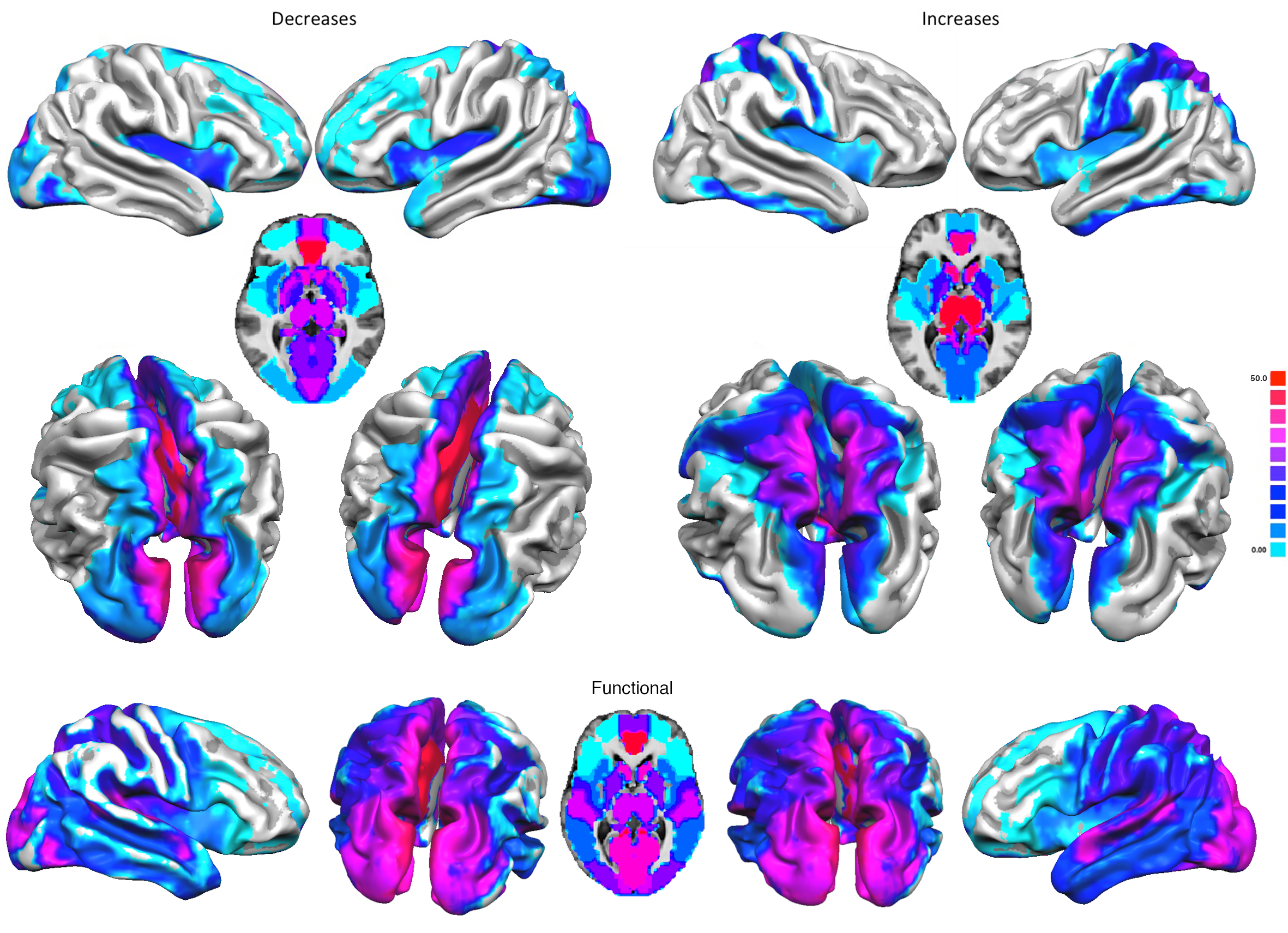


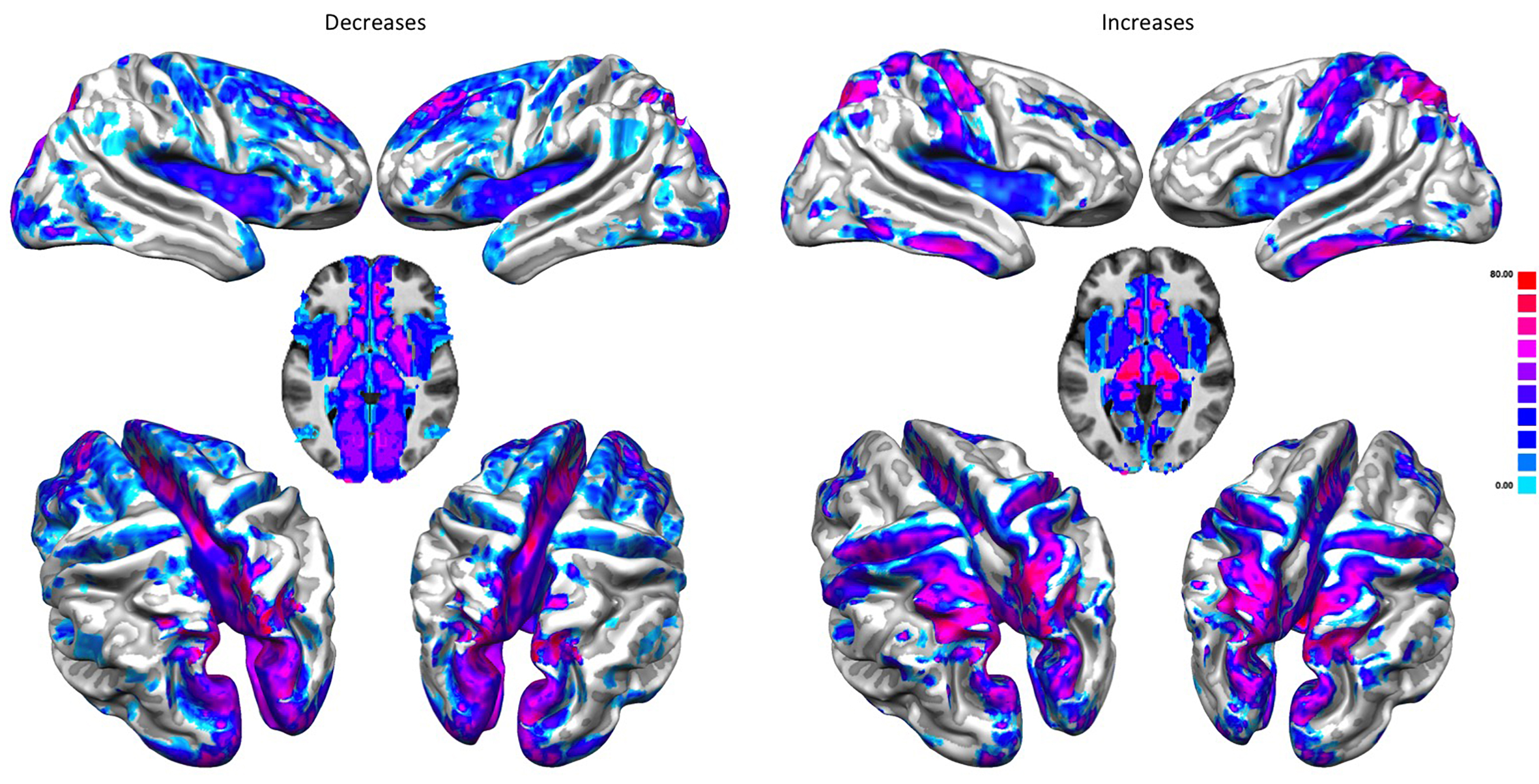
**Figure S3:** As a comparison with Figure S2, the pathological homotopic anatomical co-alteration (PHAC) for decreases and increases obtained with the Talairach atlas.

**Figure S4:** Reliability towards subsampling of the edges showing significant homotopic co-alteration in PHAC and dPHAC maps. Standard error values were multiplied by 100 for visualization purpose.

**
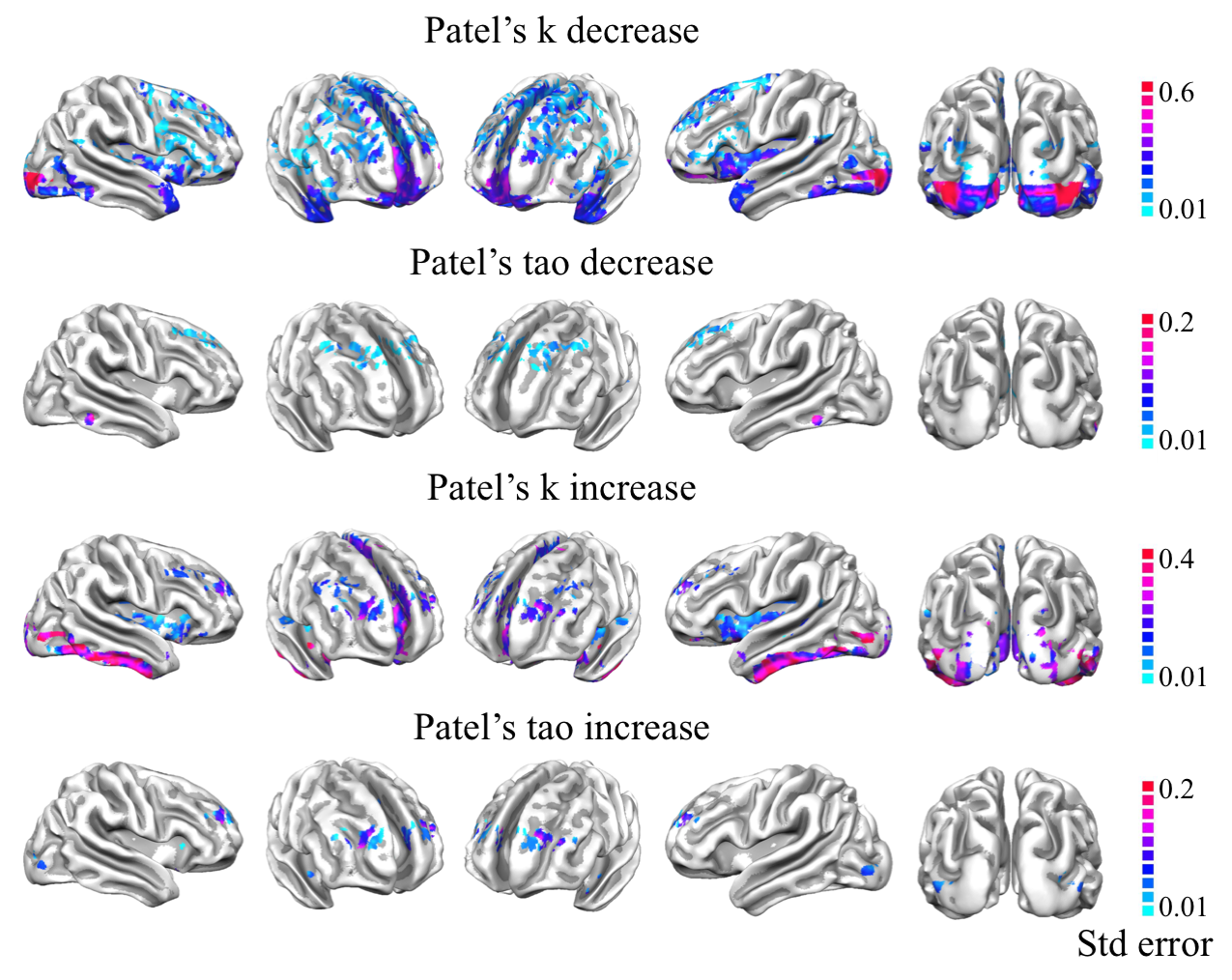
**

**Table S1**. Description of the total voxel-based morphometry experimental sample with respective diagnostic labeling (ICD-10 code).

| ***ICD-10 Code*** | ***Diagnosis*** | ***Subjects***  ***(Number)*** | ***Experiments***  ***(Number)*** | |
| --- | --- | --- | --- | --- |
|  |  |  | ***GM Decrease*** | ***GM Increase*** |
| **B10** | Other Human Herpesviruses | 8 | 1 | 0 |
| **C71** | Malignant Neoplasm of Brain | 42 | 2 | 1 |
| **C91** | Lymphoblastic Leukemia | 13 | 1 | 0 |
| **D57** | Sickle-Cell Disease | 36 | 2 | 0 |
| **E10/E11** | Diabetes mellitus | 207 | 3 | 7 |
| **E23** | Hypofunction and Other Disorders of the Pituitary Gland | 96 | 3 | 3 |
| **E66** | Obesity | 48 | 1 | 1 |
| **F10** | Alcohol Related Disorders | 140 | 9 | 0 |
| **F15** | Other Stimulant Related Disorders | 127 | 3 | 1 |
| **F20** | Schizophrenia | 4944 | 114 | 33 |
| **F29** | Psychosis | 697 | 20 | 13 |
| **F31** | Bipolar Disorder | 1910 | 49 | 27 |
| **F32/F33** | Major/Single Episode/Recurrent Depressive Disorder | 3248 | 90 | 21 |
| **F41** | Other Anxiety Disorders | 250 | 10 | 4 |
| **F42** | Obsessive Compulsive Disorder | 739 | 16 | 11 |
| **F43** | Reaction to Severe Stress and Adjustment Disorders | 476 | 30 | 2 |
| **F84** | Pervasive Developmental Disorders | 1499 | 37 | 37 |
| **F50** | Eating Disorders | 254 | 14 | 7 |
| **F60** | Specific Personality Disorders | 120 | 7 | 0 |
| **F65** | Paraphilias | 59 | 4 | 0 |
| **F79** | Mental Retardation | 1015 | 57 | 8 |
| **F80** | Specific Developmental Disorders of Speech and Language | 34 | 2 | 2 |
| **F88** | Other Disorders of Psychological Development | 114 | 3 | 4 |
| **F90** | Attention Deficit/Hyperactivity Disorder | 181 | 10 | 2 |
| **F91** | Conduct Disorder | 359 | 7 | 6 |
| **G10** | Huntington's Disease | 279 | 12 | 1 |
| **G11** | Hereditary Ataxia | 191 | 12 | 1 |
| **G12** | Spinal Muscular Atrophy and Related Syndromes | 339 | 22 | 1 |
| **G20** | Parkinson's Disease | 1041 | 46 | 13 |
| **G23** | Progressive Supranuclear Palsy | 252 | 14 | 2 |
| **G24** | Dystonia | 222 | 6 | 8 |
| **G30** | Alzheimer’s Disease | 1297 | 61 | 22 |
| **G31** | Other Degenerative Diseases of Nervous System | 896 | 38 | 22 |
| **G35** | Multiple Sclerosis | 1874 | 54 | 11 |
| **G40** | Epilepsy and Recurrent Seizures | 2414 | 67 | 26 |
| **G43** | Migraines | 150 | 7 | 0 |
| **G44** | Idiopathic Headache Disorder | 261 | 6 | 5 |
| **G47** | Sleep Disorders | 294 | 11 | 5 |
| **G50** | Disorders of Trigeminal Nerve | 140 | 3 | 1 |
| **G90** | Disorders of Autonomic Nervous System | 15 | 1 | 0 |
| **G93** | Other Disorders of Brain | 83 | 5 | 2 |
| **H53** | Visual Disturbances blepharospasmus | 72 | 1 | 2 |
| **H54** | Blindness and Low Vision | 25 | 2 | 0 |
| **H55** | Nystagmus and other Irregular Eye Movements | 97 | 3 | 3 |
| **H81** | Disorders of Vestibular Function | 30 | 1 | 1 |
| **H90** | Conductive and Sensorineural Hearing Loss | 10 | 0 | 1 |
| **H93** | Other Disorders of ear, Not Elsewhere Classified | 28 | 1 | 0 |
| **I63** | Cerebral Infarction | 10 | 1 | 0 |
| **K58** | Irritable Bowel Syndrome | 107 | 2 | 1 |
| **L59** | Other Disorders of Skin and Subcutaneous Tissue Related to Radiation | 40 | 4 | 0 |
| **M19** | Other and Unspecified Osteoarthritis | 182 | 5 | 6 |
| **M26** | Dentofacial Anomalies | 40 | 0 | 2 |
| **M54** | Dorsalgia | 254 | 5 | 3 |
| **M79** | Other and Unspecified Soft Tissue Disorders, Not Elsewhere Classified | 466 | 21 | 5 |
| **N94** | Pain and Other Conditions Associated with Femal Genital Organs and Menstrual Cycle | 132 | 3 | 4 |
| **P07** | Disorders of Newborn Related to Short Gestation and Low Birth Weight, Not Elsewhere Classified | 312 | 10 | 4 |
| **Q04** | Other Congenital Malformations of Brain | 123 | 1 | 10 |
| **Q90** | Down Syndrome | 22 | 1 | 1 |
| **Q93** | Monosomies and Deletions from Autosomes, Not Elsewhere Classified | 167 | 10 | 4 |
| **Q96** | Turner’s Syndrome | 104 | 4 | 4 |
| **R27** | Other Lack of Coordination | 42 | 1 | 4 |
| **R43** | Disturbances of Smell and Taste | 22 | 1 | 0 |
| **R47** | Speech Disturbances, Not Elsewhere Classified | 241 | 19 | 9 |
| **R48** | Dyslexia and Other Symbolic Dysfunctions, Not Elsewhere Classified | 144 | 9 | 3 |
| **R53** | Malaise and Fatigue | 16 | 1 | 0 |
| **R55** | Syncope and Collapse | 92 | 1 | 2 |
| **R90** | Abnormal Findings on Diagnostic Imaging of Central Nervous System | 53 | 2 | 0 |
| **S06** | Intracranial Injury | 82 | 5 | 0 |
| **S24** | Injury of Nerves and Spinal Cord at Thorax Level | 27 | 2 | 0 |
| **T76** | Adult and Child Abuse, Neglect and Other Maltreatment, Suspected | 43 | 2 | 2 |
| **Z89** | Acquired Absence of Limb | 56 | 2 | 0 |
| ***TOTAL*** | | ***29403*** | ***980*** | ***381*** |

**Table S2**. Sample characteristics and gray matter (GM) variations with relative numbers of foci for each of the four most represented brain disorders in the BrainMap voxel-based morphometry database.

Experiments (N) = number of experiments; Experiments (%) = percentage of the total of the selected experiments; Subj (N) = number of subjects.

| ***Diagnosis*** | ***Articles*** | | ***Experiments*** | | ***Subj (N)*** | ***GM Changes*** | |
| --- | --- | --- | --- | --- | --- | --- | --- |
|  | ***Decrease*** | ***Increase*** | ***(N)*** | ***(%)*** |  | ***Decrease*** | ***Increase*** |
| Schizopherenia | 83 | 31 | 147 | 10.8 | 4944 | 1481 | 273 |
| Alzheimer’s disease | 44 | 11 | 83 | 6.1 | 1297 | 818 | 143 |
| Bipolar disorder | 34 | 20 | 76 | 5.6 | 1910 | 254 | 107 |
| Depressive disorder | 34 | 12 | 111 | 8.1 | 3248 | 430 | 60 |
| ***TOTAL*** | **195** | **74** | **417** | **30.6** | **11399** | **2983** | **583** |

**Table S3**. Types of paradigm classes with relative number of experiments and subjects for each paradigm class of the functional BrainMap database used in the analysis.

Experiments (N) = number of experiments; Experiments (%) = percentage of the total of the selected experiments; Subj (N) = number of subjects.

| ***Paradigm Class*** | ***Articles*** | ***Experiments*** | | ***Subj (N)*** |
| --- | --- | --- | --- | --- |
|  |  | ***(N)*** | ***(%)*** |  |
| Acupuncture | 7 | 33 | 0.3 | 193 |
| Affective pictures | 33 | 225 | 1.7 | 1326 |
| Affective words | 8 | 62 | 0.5 | 398 |
| Anti Saccades | 8 | 31 | 0.2 | 202 |
| Chewing/swallowing | 15 | 90 | 0.7 | 397 |
| Classical conditioning | 17 | 90 | 0.7 | 377 |
| Competition/cooperation | 3 | 29 | 0.2 | 116 |
| Counting/calculation | 44 | 232 | 1.8 | 1116 |
| Cued Explicit Recognition/recall | 65 | 382 | 2.9 | 1791 |
| Deception | 12 | 52 | 0.4 | 340 |
| Delay discounting | 5 | 42 | 0.3 | 262 |
| Dealyed match to sample | 48 | 259 | 2.0 | 1397 |
| Divided auditory attention | 4 | 24 | 0.2 | 98 |
| Drawing | 4 | 39 | 0.3 | 77 |
| Driving | 3 | 15 | 0.1 | 69 |
| Emotion induction | 81 | 462 | 3.5 | 2790 |
| Emotional body language perception | 2 | 17 | 0.1 | 51 |
| Encoding | 48 | 236 | 1.8 | 1489 |
| Episodic recall | 22 | 117 | 0.9 | 554 |
| Estimation | 4 | 15 | 0.1 | 120 |
| Face monitoring/discrimination | 97 | 573 | 4.4 | 2755 |
| Figurative language | 6 | 33 | 0.3 | 160 |
| Film viewing | 42 | 266 | 2.0 | 1199 |
| Finger tapping/button press | 193 | 1177 | 9.0 | 7011 |
| Fixation | 9 | 30 | 0.2 | 185 |
| Flanker | 10 | 37 | 0.3 | 266 |
| Flashing checkerboard | 3 | 33 | 0.3 | 185 |
| Flexion/extension | 34 | 199 | 1.5 | 790 |
| Fluency induction | 1 | 3 | 0.0 | 12 |
| Free list word record | 4 | 11 | 0.1 | 69 |
| Gambling | 22 | 159 | 1.2 | 702 |
| Go/No go | 50 | 246 | 1.9 | 1841 |
| Grasping | 9 | 45 | 0.3 | 146 |
| Hand-Eye Coordination | 2 | 7 | 0.1 | 44 |
| Hunger/satiety | 6 | 26 | 0.2 | 134 |
| HyperCapnia /air Hunger | 7 | 22 | 0.2 | 104 |
| Imagined movement | 20 | 94 | 0.7 | 522 |
| Imagined objects/scenes | 25 | 128 | 1.0 | 742 |
| Induced panic | 2 | 6 | 0.0 | 62 |
| Isometric force | 5 | 26 | 0.2 | 104 |
| Lexical decision | 6 | 26 | 0.2 | 165 |
| Magnitude comparison (distance) | 1 | 15 | 0.1 | 38 |
| Magnitude comparison (luminance) | 2 | 13 | 0.1 | 37 |
| Magnitude comparison (numerical) | 2 | 13 | 0.1 | 37 |
| Magnitude comparison (phisical size) | 3 | 20 | 0.2 | 97 |
| Magnitude comparison (symbolic) | 5 | 24 | 0.2 | 126 |
| Meditation | 11 | 85 | 0.6 | 433 |
| Mental rotation | 19 | 117 | 0.9 | 493 |
| Micturition | 6 | 25 | 0.2 | 128 |
| Motor learning | 1 | 7 | 0.1 | 15 |
| Multi tasking | 3 | 14 | 0.1 | 82 |
| Music comprehension | 19 | 155 | 1.2 | 510 |
| Music production | 17 | 77 | 0.6 | 407 |
| N-back | 64 | 291 | 2.2 | 2609 |
| Naming (covert) | 14 | 73 | 0.6 | 288 |
| Naming ( overt) | 20 | 120 | 0.9 | 467 |
| Object manipulation/discrimination | 4 | 32 | 0.2 | 58 |
| Oddball discrimination | 12 | 48 | 0.4 | 376 |
| Olfactory monitoring/discrimination | 22 | 141 | 1.1 | 557 |
| Orthographic discrimination | 24 | 124 | 0.9 | 653 |
| Pain monitor/discrimination | 63 | 325 | 2.5 | 1439 |
| Paired associate recall | 27 | 138 | 1.0 | 699 |
| Passive listening | 48 | 234 | 1.8 | 1153 |
| Passive viewing | 79 | 450 | 3.4 | 2186 |
| Phonological discrimination | 38 | 209 | 1.6 | 857 |
| Pitch monitor/discrimination | 22 | 142 | 1.1 | 554 |
| Pointing | 8 | 34 | 0.3 | 113 |
| Pursuit rotor/manual tracking | 4 | 21 | 0.2 | 75 |
| Reading (covert) | 37 | 194 | 1.5 | 872 |
| Reading (overt) | 26 | 131 | 1.0 | 595 |
| Reasoning/problem solving | 36 | 206 | 1.6 | 1318 |
| Recitation/repetition (covert) | 10 | 31 | 0.2 | 212 |
| Recitation/repetition (overt) | 19 | 78 | 0.6 | 408 |
| Rest | 11 | 36 | 0.3 | 386 |
| Reward | 106 | 728 | 5.5 | 3432 |
| Saccades | 31 | 123 | 0.9 | 574 |
| Self reflection | 2 | 24 | 0.2 | 87 |
| Semantic monitor/discrimination | 109 | 616 | 4.7 | 2960 |
| Sequence recall/learning | 14 | 65 | 0.5 | 334 |
| Sexual arousal/gratification | 17 | 122 | 0.9 | 609 |
| Sleep | 3 | 7 | 0.1 | 77 |
| Stroop - color | 30 | 110 | 0.8 | 1049 |
| Stroop - counting | 4 | 14 | 0.1 | 105 |
| Stroop - emotional | 8 | 23 | 0.2 | 328 |
| Stroop - other | 3 | 14 | 0.1 | 103 |
| Stroop - spatial | 2 | 10 | 0.1 | 36 |
| Syntactic discrimination | 8 | 23 | 0.2 | 212 |
| Tactile monitor/discrimination | 28 | 134 | 1.0 | 580 |
| Task switching | 22 | 117 | 0.9 | 644 |
| Taste | 18 | 113 | 0.9 | 623 |
| Theory of mind | 38 | 253 | 1.9 | 1197 |
| Thirst induction | 3 | 11 | 0.1 | 67 |
| Tone monitor/discrimination | 27 | 119 | 0.9 | 643 |
| Tower of London | 4 | 15 | 0.1 | 84 |
| Transcranical Magnetic Stimulation | 8 | 35 | 0.3 | 129 |
| Trauma recall | 2 | 7 | 0.1 | 68 |
| Vestibular Stimulation | 3 | 7 | 0.1 | 54 |
| Vibrotactile monitor/discrimination | 8 | 130 | 1.0 | 169 |
| Video games | 4 | 20 | 0.2 | 111 |
| Visual Motion | 2 | 11 | 0.1 | 55 |
| Visual object identification | 33 | 229 | 1.7 | 853 |
| Visual pursuit/tracker | 19 | 95 | 0.7 | 390 |
| Visuospatial attention | 70 | 353 | 2.7 | 1693 |
| Wisconsin card sorting test | 12 | 57 | 0.4 | 352 |
| Word generation (covert) | 35 | 155 | 1.2 | 779 |
| Word generation (overt) | 28 | 142 | 1.1 | 782 |
| Word imageability | 3 | 13 | 0.1 | 84 |
| Word stem completion (covert) | 2 | 6 | 0.0 | 64 |
| Word Stem completion (overt) | 4 | 14 | 0.1 | 103 |
| Writing | 3 | 11 | 0.1 | 83 |
| ***TOTAL*** | ***2376*** | ***13148*** | ***100%*** | ***68152*** |
